# Solvothermal synthesis and structural characterization of three polyoxotitanium-organic acid clusters†

**DOI:** 10.1039/d0ra09691a

**Published:** 2021-07-23

**Authors:** Katarzyna Kazimierczuk, Marco Milanesio, Anna Dołęga, Luca Palin, Maja Walencik, Michał Jurkowski, Eleonora Conterosito

**Affiliations:** Department of Inorganic Chemistry, Chemical Faculty, Gdańsk University of Technology Narutowicza St. 11/12 80-233 Gdańsk Poland; Dipartimento di Scienze e Innovazione Tecnologica Via T. Michel 11 15121 Alessandria Italy eleonora.conterosito@uniupo.it; Nova Res s.r.l. Via D. Bello 3 28100 Novara Italy https://www.novares.org

## Abstract

Three new titanium oxo-clusters Ti_4_O_2_(O^i^Pr)_10_(OOCPhMe)_2_ (I), Ti_6_O_4_(OEt)_8_(OOCPhMe)_8_ (II) and Ti_6_O_6_(OEt)_6_(OOCCHPh_2_)_6_ (III) were obtained by easy one-step solvothermal reactions of titanium(iv) isopropoxide, alcohols and carboxylic acids. The three compounds were characterized by single-crystal and powder X-ray diffraction, TGA/DSC, optical and electron microscopy, and FTIR and NMR spectroscopy. X-ray powder diffraction and spectroscopy confirmed the purity of the compounds. Structural analysis indicates that in all compounds the titanium(iv) ions are six-coordinated (distorted octahedra). (I) is a tetranuclear complex containing a Ti_4_(μ_4_-O)(μ_2_-O) core, which is linked by two (μ_2_-OOCPhMe), four (μ_2_-O^i^Pr) and six O^i^Pr ligands. (II) and (III) are hexanuclear complexes with different cores, respectively Ti_6_(μ_3_-O)_2_(μ_2_-O)_2_ and Ti_6_(μ_3_-O)_6_. The coordination sphere of the Ti atoms is filled by eight (μ_2_-OOCPhMe), two (μ_2_-OEt) and six OEt in (II) and six (μ_2_-OOCHPh_2_) and six OEt in (III). Different steric hindrance of substituents attached to the carboxyl group or different concentrations lead to three main different cluster geometries with two ligands. The tetranuclear and the hexanuclear clusters were obtained with the OOCPhMe ligand, while the hexagonal prism cluster was obtained with the OOCCHPh_2_ ligand. Hirshfeld surface calculations indicated that the packing is driven by C–O⋯H–C weak hydrogen bonds. The clusters can be used as molecular models of organic molecules bonded to titania surface, used in organic photovoltaic (dye sensitized solar cells) or other optoelectronic applications.

## Abstract 2

Crystal structures of three novel titanium oxo-clusters with different cores and ligands obtained by an easy and convenient solvothermal method.

## Graphical abstract

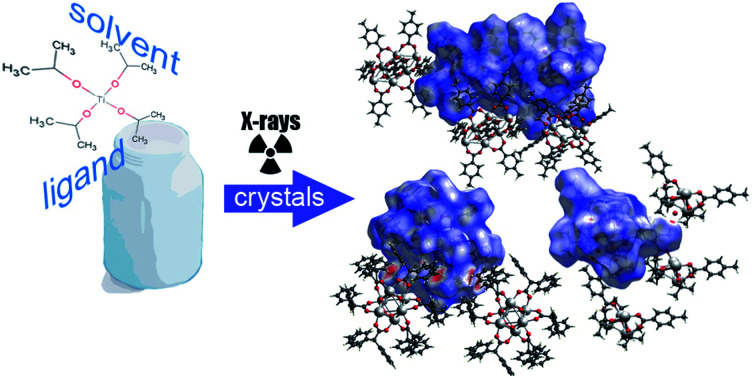

## Introduction

Titanium oxo clusters (TOCs) are widely studied for catalysis applications and as building blocks for metal–organic frameworks and some of them exhibit CO_2_ adsorption properties.^1–5^ Moreover, ligand functionalized TOCs can be used as models to study the physical and surface properties of the molecules bonded to titania surfaces. Among the many related fields of applications, the more relevant examples are related to dye sensitized TiO_2_ nanoparticles^6,7^ used in dye sensitized solar cells (DSSCs),^8–10^ and TiO_2_-induced photodegradation of pollutants.^11–13^ In fact, recently a number of titanium oxo-clusters have been synthesized with photoactive ligands^14^ and characterized with the aim of studying the synergistic effect between the titanium core and the ligands envisioning possible applications in photocatalysis^15^ and DSSCs^16,17^ thanks also to the possibility of tuning the band gap of TiO_2_ by sensitization with dyes.^18^

The study of the packing and interactions between the molecules allows shedding light, for instance, on the charge transfer properties^19^ and on structure dependent quenching or enhancement of fluorescence in photoactive compounds.^20,21^ Recently, it was reported that also some small phosphinate supported Ti-oxo clusters show photoactivated charge separation and can be useful as case study for the development of efficient photocatalytic processes.^11^

In this study, clusters with Ti(iv) and *p*-toluic acid or diphenylacetic acid as ligand were synthesized, namely Ti_4_O_2_(O^i^Pr)_10_(OOCPhMe)_2_ (I), Ti_6_O_4_(OEt)_8_(OOCPhMe)_8_ (II) and Ti_6_O_6_(OEt)_6_(OOCCHPh_2_)_6_ (III) by easy, one-step solvothermal reactions.^22^ By changing the stoichiometry of reactants during the synthesis we were able to obtain two different clusters with the same ligand but with different types of cores and another cluster with a different Ti–O core structure using another ligand^23,24^ Ti–O clusters with *p*-toluic acid were never reported up to now but, more interestingly, the observed Ti_4_(μ_4_-O) core is not very common.^6,25,26^ The crystal structures of the three clusters were solved and refined by single crystal diffraction analysis and the crystal packing was studied and compared exploiting the Hirshfeld surface approach. All clusters were characterized by infrared (IR) and diffused reflectance UV-Vis (DR UV-Vis) spectroscopies, X-ray powder diffraction (XRPD), thermogravimetrical analysis (TGA and DSC), NMR spectroscopy, optical and scanning electron microscopy (SEM).

## Results and discussion

Ti-oxo-carboxylate clusters with general formula Ti_*n*_O_*m*_(OR)_*x*_(OOCR′)_*y*_ (R = ^i^Pr, Et; R′ = PhMe, CHPh_2_, C_5_H_4_N) were synthesized from the reaction of titanium(iv) tetraisopropoxide (Ti(O^i^Pr)_4_) as metal cation precursor with the organic acids. Crystalline materials for (I)–(III) were obtained successfully by solvothermal synthesis carried out in EtOH or ^i^PrOH at mild temperatures of 80–90 °C.

X-ray diffraction studies proved that in all three structures, each titanium atom is coordinated to six O atoms in a distorted octahedral geometry. The three titanium-oxo clusters have increasing condensation degrees^27^ (O/Ti) of 0.33 for (I), 0.56 for (II) and 1 for (III) which are determined by the hydrolysis ratio and influenced by the concentration of reactants since the carboxylic acid serves not only as a ligand but also as a source of water through esterification.

**Scheme 1:**
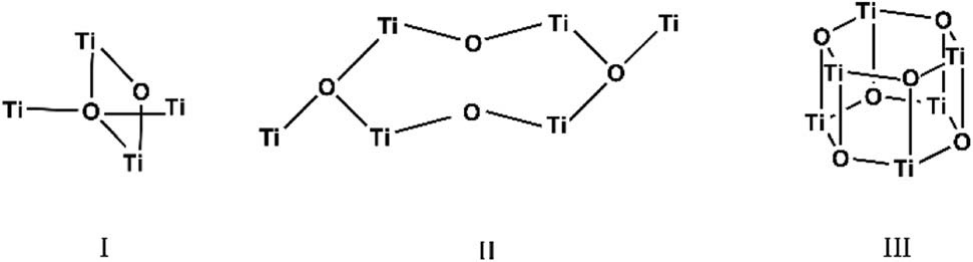
Representation of the Ti–O cores structures of the three compounds.

The carboxylate anions of organic acids form the typical bridges between two Ti atoms (Scheme 1), and isopropoxide ligands complete the coordination sphere of the metal atoms. The three structures are hereafter discussed highlighting peculiarities and similarities of the three clusters. The crystallographic data are reported in Table SI1.†

### IR and NMR spectroscopy

**Fig. 1:**
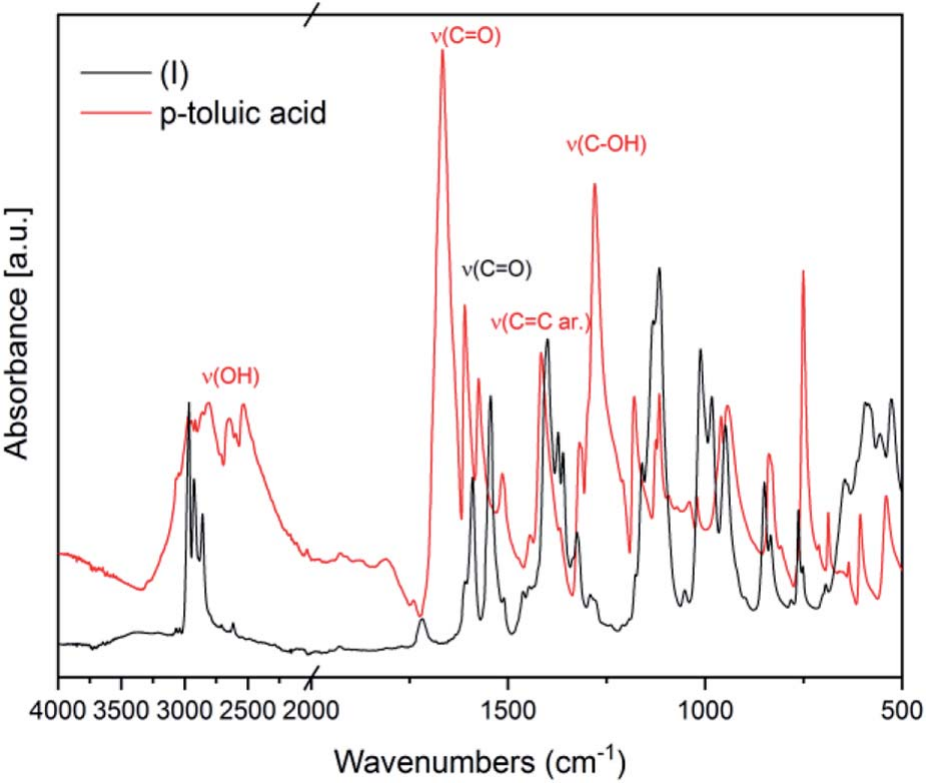
FT-IR spectra of (I) and of the corresponding ligand *p*-toluic acid.

IR spectroscopy was used to confirm the cluster formation and investigate the effects of the binding of the organic molecules with the titanium oxide moiety. In Fig. 1 the IR spectra of (I) is shown along with the corresponding spectrum of the *p*-toluic acid ligand alone for comparison. The broad band between 2500–3200 cm^−1^ attributable to OH stretching is visible in the spectrum of the *p*-toluic acid and not in the spectrum of the cluster (I), indicating the complete reaction of the ligand. The strong band attributable to C=O stretching is shifted toward lower wavenumbers in the cluster indicating a weaker bond, while the band attributed to C–OH stretching is not present in the spectra of (I) confirming once more the coordination. In the fingerprint region of (I) there are three new bands at 555, 649 and 984 cm^−1^ that can be attributed to the formation of the complex with titanium.

**Fig. 2:**
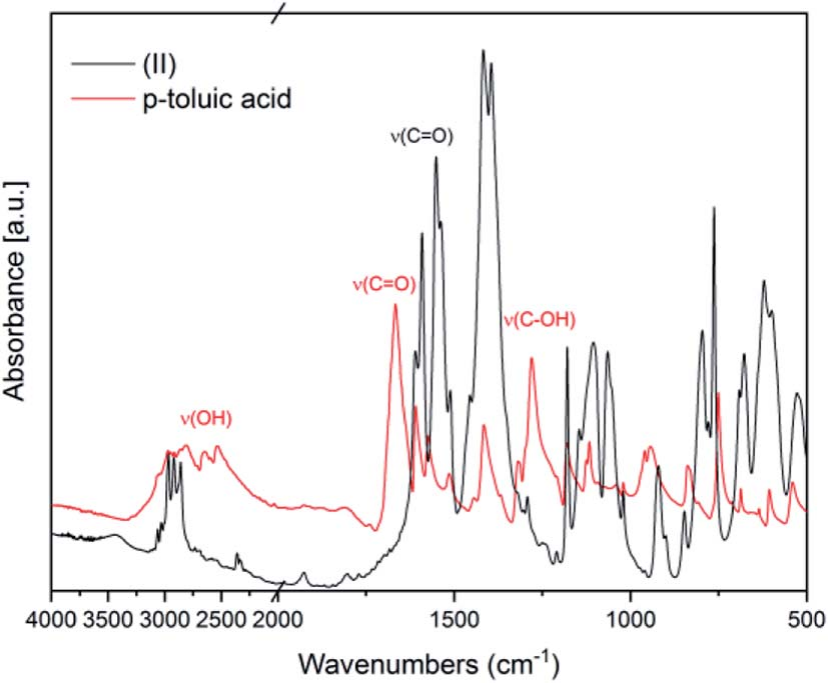
FT-IR spectra of (II) and of the ligand *p*-toluic acid

In Fig. 2 FT-IR spectra of (II) and of the ligand *p*-toluic acid are reported. Also in this case, the disappearance of the OH band, the shift of the C=O band and the absence of the C–OH stretching band confirm the formation of the desired product.

**Fig. 3:**
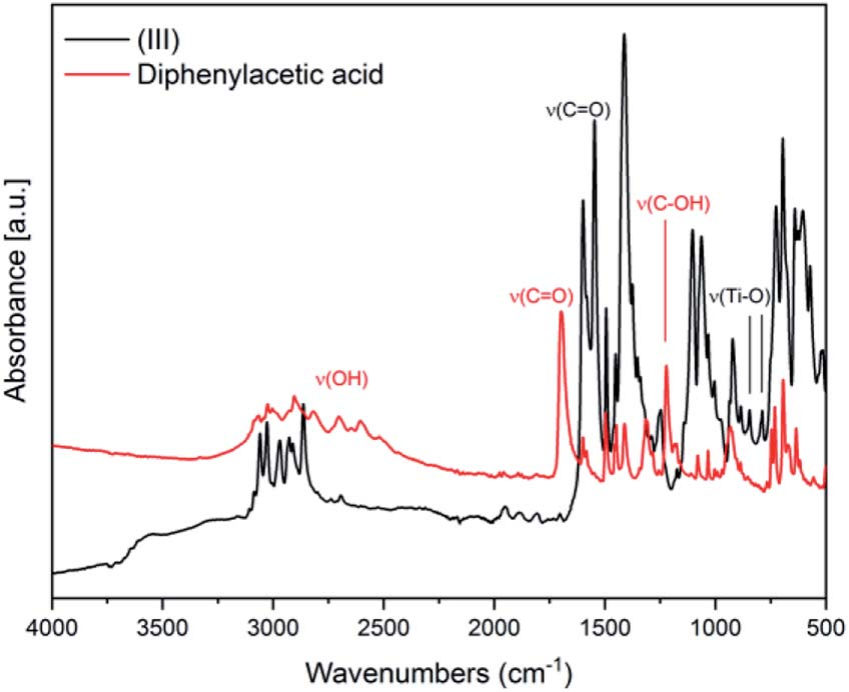
FT-IR spectra of (III) and of the ligand diphenyl acetic acid.

In Fig. 3 the IR spectra of (III) is reported along with the corresponding spectrum of the ligand for comparison. The broad band between 2500–3200 cm^−1^ attributable to OH stretching, visible in the spectrum of diphenylacetic acid, is no longer present in the spectra of the complex, indicating the complete coordination of diphenylacetic ligands. The band attributable to C=O stretching falling at 1697 cm^−1^ in the spectra of diphenylacetic acid alone is split and shifted to lower wavenumbers in the spectra of the complex. In the fingerprint region, the changes in the structure due to the coordination to titanium are evidenced by the appearance of two new bands at 787 and 843 cm^−1^ that can be attributed to Ti–O stretching.^28^

The IR data confirm that the products are pure.

NMR spectra for (I) are shown in ESI as Fig. SI8 and SI9.† All the compounds are poorly or very poorly soluble in common organic solvents and the spectra were measured for samples prepared as suspensions in DMSO-d6. Only for complex (I) we were able to register some peaks characteristic for 2-propanol but none for toluic acid, which in our opinion indicates that in DMSO complex (I) underwent decomposition.

### DR UV-Vis spectroscopy analysis

**Fig. 4:**
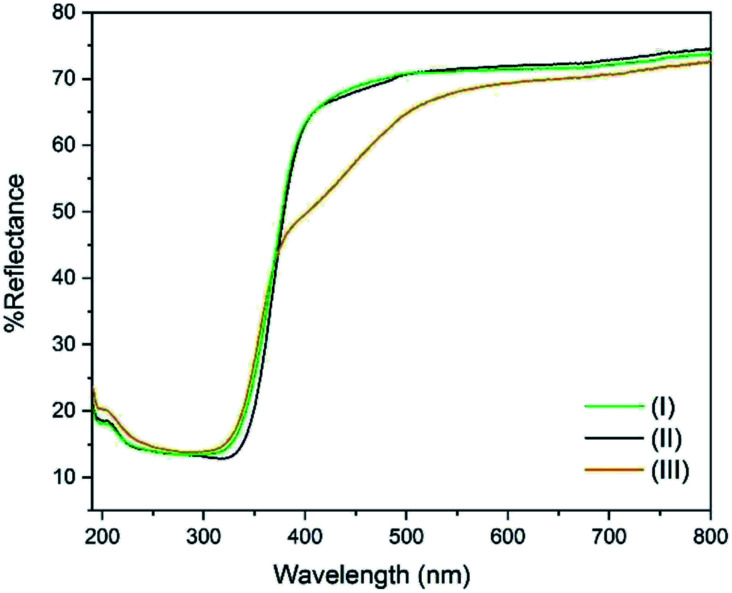
DR-UV-Vis spectra of compounds (I) (green) (II), (black), and (III) (orange).

The DR UV-Vis spectra of the three compounds were collected and are shown in Fig. 4. It can be seen that the band gap is identical (3.36 eV) for (I) and (II) which share the same ligand (*p*-toluic acid). Conversely (III), shows a more complex band gap. Its reflectance is identical to (I) and (II) below 360 nm, but a marked shoulder in the visible region is evident with the band centred at 455 nm. In fact (III), is slightly yellowish. We can conclude that the yellow absorption is not due to superficial impurities or degradation since it does not change when grinding the crystals. A band gap in the visible of 2.72 eV can be calculated, even if the ligand alone does not absorb in this region. Such behaviour was already observed in planar Ti clusters bound to 1-hydroxy-2-naphthoic acid by Ding *et al.*^29^

### TGA-DSC analysis

TGA-DSC analysis were carried out to investigate the thermal stability and decomposition behaviour of the three compounds. The analysis were carried out under N_2_ flux (simulating the environment of photovoltaic and LED product where photoactive materials are usually employed) from ambient temperature to 600 °C. Compound I exhibits a first weight loss between 150 and 170 °C (∼11%) and a second larger one at 214.58 °C (∼48%) (Fig. SI2†) that can be attributed to the loss of the iPrOH residues (at first the one interacting with one Ti atom and then the one bridging between two Ti atoms) as confirmed by the endothermic DSC peaks (Fig. SI2†). Between 290° and 300 °C there is the last weight loss of about 10% associated to an endothermic peak. The final residue amount (21.5%) agrees with expected residual TiO_2_ content calculated by the crystal structure (20.6%) (Table SI7†). The behaviour of (II) (Fig. SI3†) and (III) (Fig. SI4†) is more complex with an initial larger stability (first weight loss at 229 and 237 °C respectively) but with most of the loss concentrated in two peaks at about 300°. (II) shows two well defined peaks and a very small residual loss at between 400 and 500°. In (III), the two peaks in the 300 °C region are more superimposed, and a much larger loss is observed in the higher T region with a marked peak at 414 °C. Looking to the structural features of the three clusters, having (I) and (II) the same ligand and (II) and (III) the same 6-member Ti ring, it is evident that the thermal stability is related more to the cluster size than to the ligand. Indeed, the organic molecules alone are stable typically up to 200–250° and it can be inferred that the decomposition starts at the organic–inorganic border and it is facilitated by the more strained 4-T ring of compound (I). Then, the ligand effect is evident at the higher temperatures, as evidenced by the differences between (II) and (III). The similarity of the behaviours of (II) and (III) is confirmed by a residual loss not in agreement with the TiO_2_ content calculated by the crystal structure (see Table SI7 and SI8†), but a bit larger, probably due to the different decomposition path occurring at larger temperatures, because of the increased stability of the organic/inorganic bond, and thus favouring the formation of stable carbon species. The heat flow from DSC reflects the differences in both cluster geometry and ligand type and is very different for the three compounds.

### XRPD analysis

XRPD patterns were collected in order to assess the purity and homogeneity of the sample by performing a Rietveld refinement (see Fig. SI5†), using the single crystal solved structures. The fit confirmed that (I) and (II) are produced as pure phases. Compound (III) formed large crystals and it is the only one in which the fit is not perfect because grinding the crystals to perform the measure caused defects that are visible by the splitting and tails of low angle peaks. The measure was repeated on a very gently ground sample, spinning during the measure, to confirm that the issue with the crystallinity is due to the grinding, but a good measure cannot be obtained in these conditions due to preferred orientations. The fit of this last XRPD pattern is shown in Fig. SI5† and spherical harmonics were used in the Rietveld fit to correct the preferred orientations. The Rietveld fit (Fig. SI5†) confirms the purity of the sample and that also in the case of (III) the single crystal is representative of the whole batch. SEM images showed few particles of titanium oxide but the percent and crystallinity are too low to be identified in the XRPD pattern.

### Microscopy

Cluster (I) and (II) show smaller crystal sizes but similar morphologies going from large to smaller crystallites, while (III) shows large crystallites mixed with smaller particles (Fig. SI6†). Since cluster (III) showed different XRPD patterns before and after grinding, SEM images were collected on the three samples and also after grinding for (III) (Fig. SI6 and SI7†), where large crystals can still be seen together with smaller ones and few spherical aggregates that are probably amorphous titanium oxide.

### Crystal structure analysis

#### Ti_4_O_2_(O^i^Pr)_10_(OOCPhMe)_2_ (I)

**Fig. 5:**
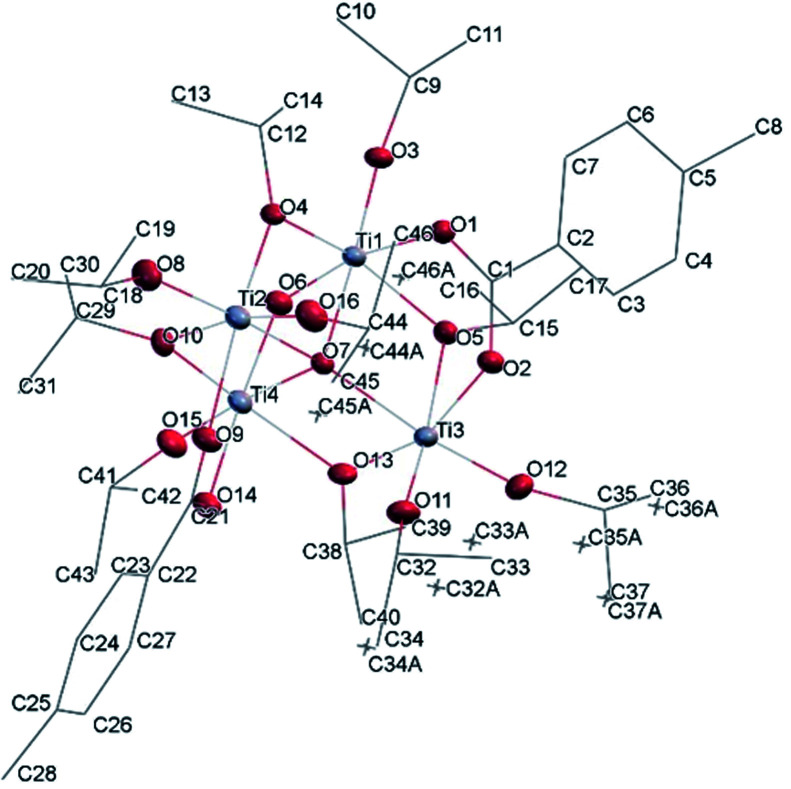
A view of the molecular structure of compound (I), with atom labelling. Displacement ellipsoids are drawn at the 50% probability level. H atoms have been omitted for clarity.

**Fig. 6:**
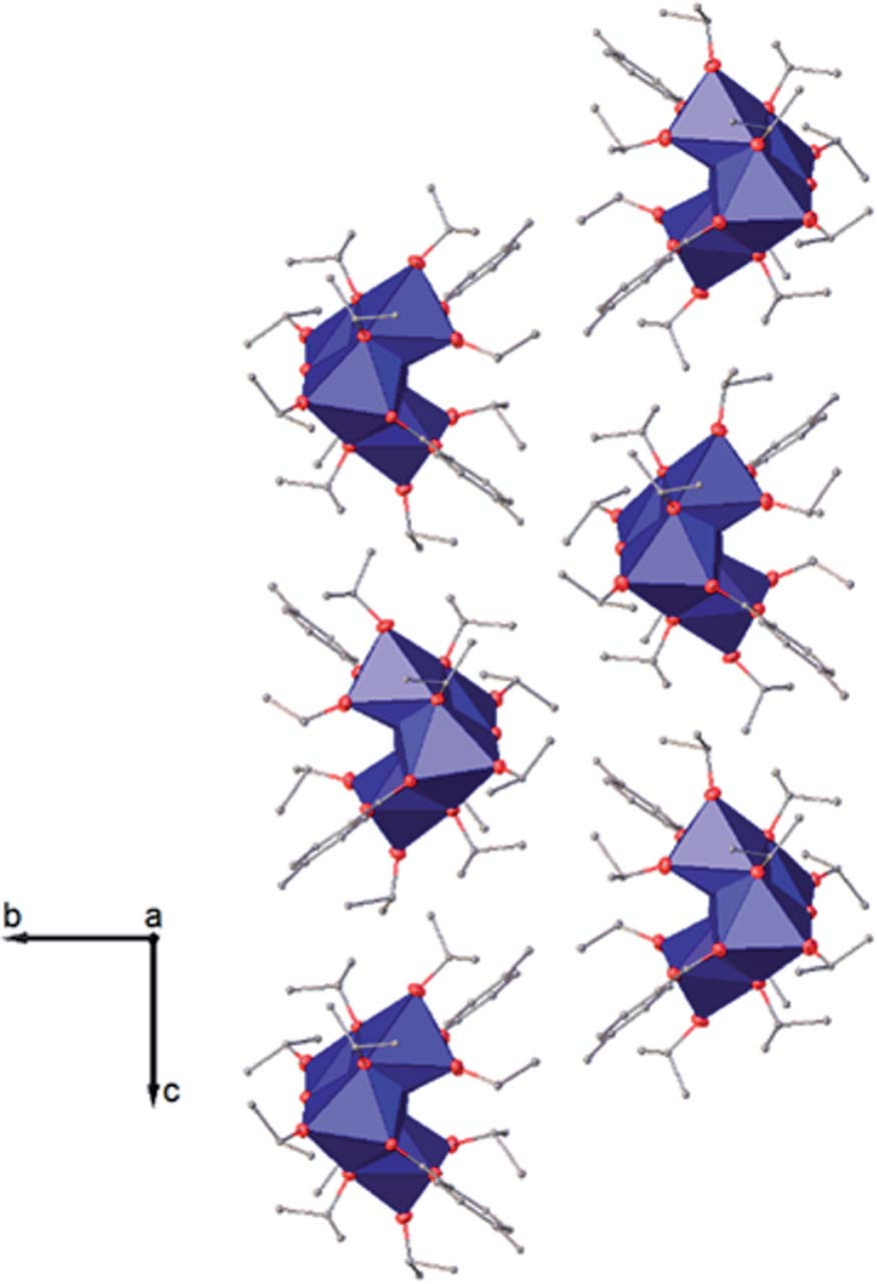
View along the *a* axis of the crystal packing of compound (I). H atoms have been omitted for clarity.

Structural analysis revealed that Ti_4_O_2_(OiPr)_10_(OOCPhMe)_2_ (I) crystallizes in the monoclinic crystal system in the *P*2_1_/*n* space group (Table SI1†). The four titanium metal centres are joined by μ_4_-O and four bridging isopropoxide ligands (μ_2_-OiPr). The degree of condensation (O/Ti) of this cluster is 0.33 while the degree of substitution (RCOO/Ti) is 0.5. The peculiarity of this cluster is the Ti_4_(μ_4_-O) core which is not very common but can be found in other clusters in crystallographic databases.^6,13,25,26,30^ By comparing (II) with these similar known clusters it can be seen that usually two carboxylic acid ligands with aromatic rings are coordinated to the core and all other ligands are smaller solvent molecules. This is due to the compact shape of this core, in fact only in one case^31^ all ligands are identical furfuryloxo moieties, where the furanic ring is one carbon atom further away from the core, reducing the steric hindrance effects. Additionally, two titanium atoms Ti(1) and Ti(4) are bound to μ_2_-O. The core arrangement is illustrated in Scheme 1a. The carboxylate anions bridge between pairs of Ti(1)–Ti(3) and Ti(2)–Ti(4) (Fig. 5). Each Ti atom becomes six coordinated in a distorted octahedral fashion by the addition of a terminal isopropoxide ligand (Fig. 5 and 6). The values of Ti–O distances and bond angles in (I) (Table SI2†) are comparable with those in complexes with similar structural features.^6,23,24^

#### Ti_6_O_4_(OEt)_8_(OOCPhMe)_8_ (II)

**Fig. 7:**
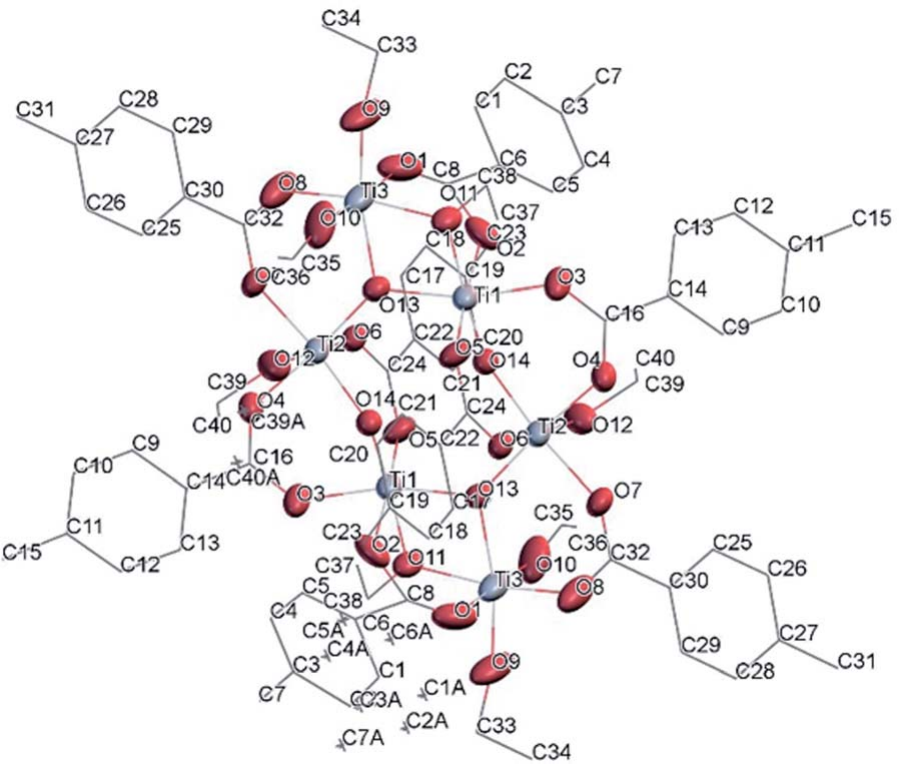
A view of the molecular structure of one of the two moieties of compound (II), with atom labelling. Displacement ellipsoids are drawn at the 50% probability level. H atoms have been omitted for clarity.

**Fig. 8:**
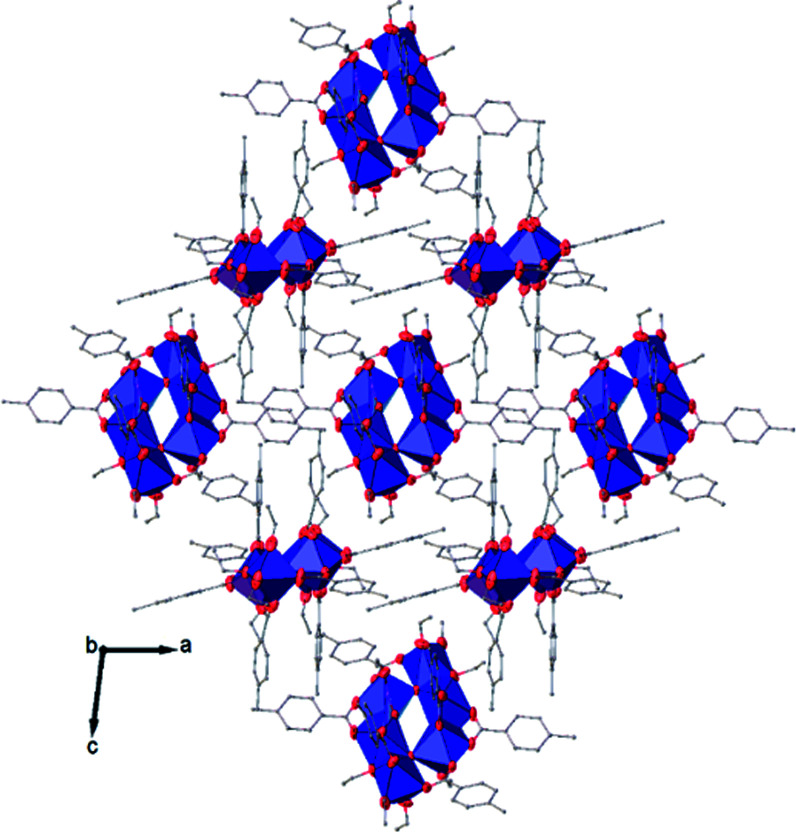
View along the *b* axis of the crystal packing of compound (II). H atoms have been omitted for clarity.

The structure of Ti_6_O_4_(OEt)_8_(OOCPhMe)_8_ (II) was solved in the triclinic space group *P*1̄ (Table SI1†). Each hexameric unit consists of two crystallographically independent halves; the second halves are generated by a centre of symmetry. The centrosymmetric Ti_6_O_4_ core is formed by two Ti_3_(μ_3_-O) units, which are connected through two μ_2_-oxygen atoms. The core arrangement is illustrated in Scheme 1b. Only the outer atoms Ti(3) and symmetry related Ti(3^#^)(−*x*, −*y*, −*z*) of the Ti_6_ core are bound by bridging (μ_2_-OEt) (Fig. 7). Additionally, Ti(1) and Ti(2) are coordinated by three bridging carboxylate ligands, and Ti(3) by two. Ti(2) and Ti(3) both fill their coordination sphere with one and two terminal OEt groups, respectively.^32^ The degree of condensation is 0.56 and the degree of substitution is 1.33. The bond distances and angles observed for (II) (Table SI3†) are consistent with literature values with the bond between the (μ_3_-O) and the Ti3 or Ti6 atom which is longer than the others Ti–O bonds.^23,32–34^ In Fig. 8 the packing of the crystal is shown.

**Fig. 9:**
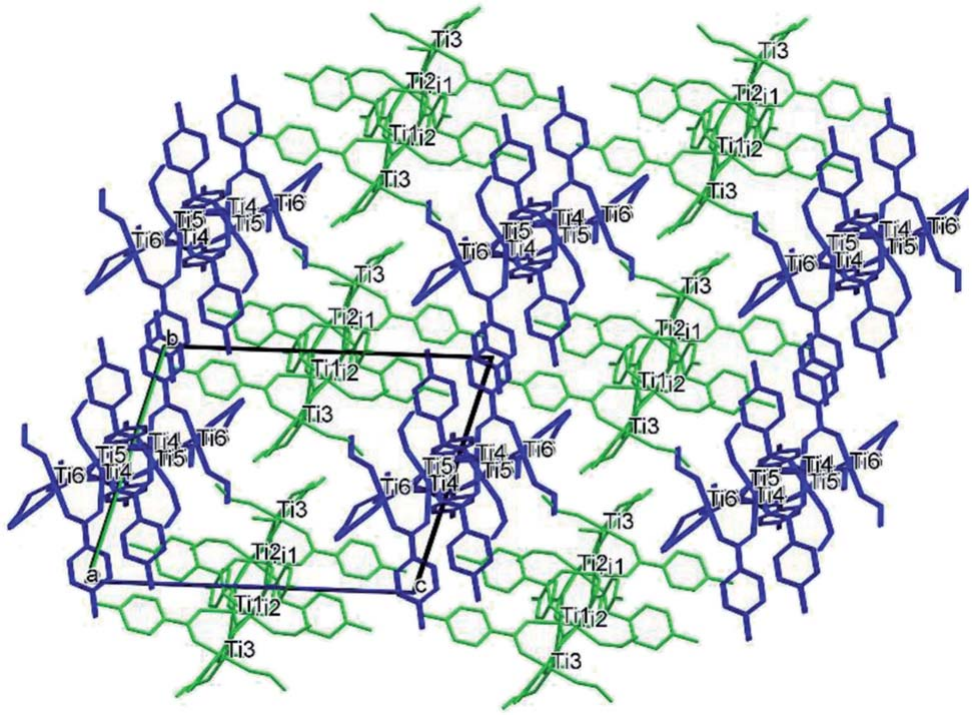
Packing of (II) viewed along the *a* axis with the molecules coloured by symmetry equivalence, the first molecule in green and the second one in blue.

**Fig. 10:**
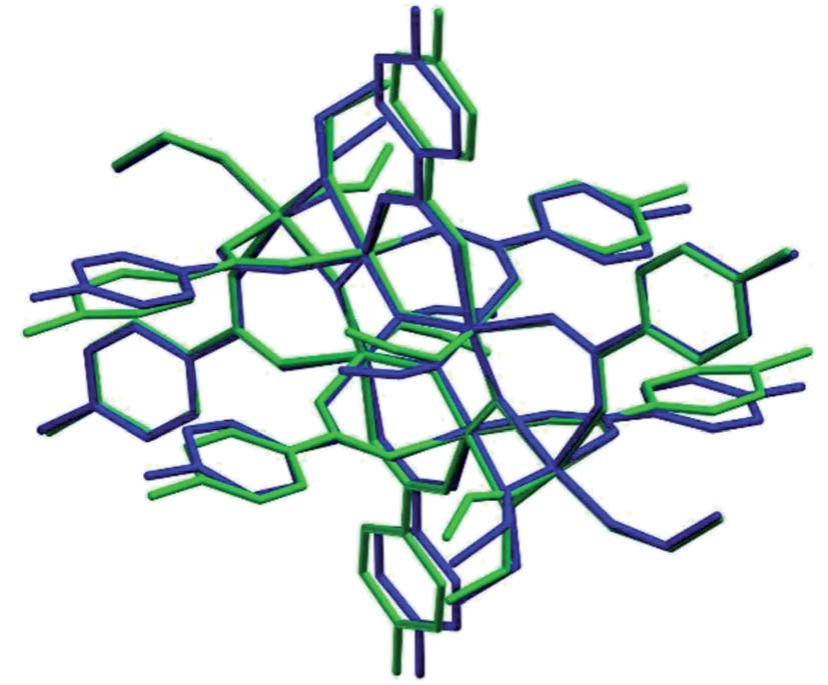
Superposition of the two moieties in (II). Hydrogens have been omitted for clarity.

**Fig. 11:**
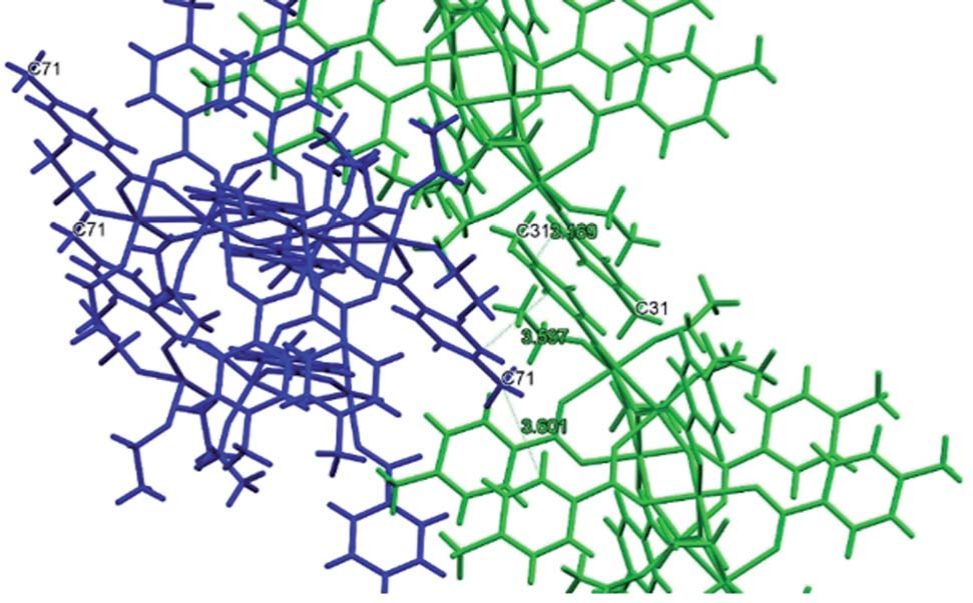
Detail of some interaction distances in the packing of (II).

The symmetry independent molecules are evidenced in the packing in Fig. 9. Despite the lack of symmetry in the packing they match almost exactly, as shown in Fig. 10. In particular, the core of the two molecules and two of the *p*-toluic ligands are almost perfectly superimposed while the other ligands form slightly different angles with the core but are in fact more mobile as indicated by the disorder and thermal ellipsoids. It is worth noting that the symmetry of the packing could ideally be higher (and the asymmetric unit made by only one half moiety). In fact, the two moieties are almost identical and their halves are specular, but they differ for their position in the packing (Fig. 9) and reciprocal interactions and they are accommodated in a triclinic centrosymmetric space group. Any attempt at solving the structure in a higher symmetry group failed, indicating that the choice of *P*1̄ is the correct one. The packing is driven by hydrophobic and π-stacking interactions and will be discussed in more detail in the Hirshfeld analysis section (Fig. 11).

#### Ti_6_O_6_(OEt)_6_(OOCCHPh_2_)_6_ (III)

**Fig. 12:**
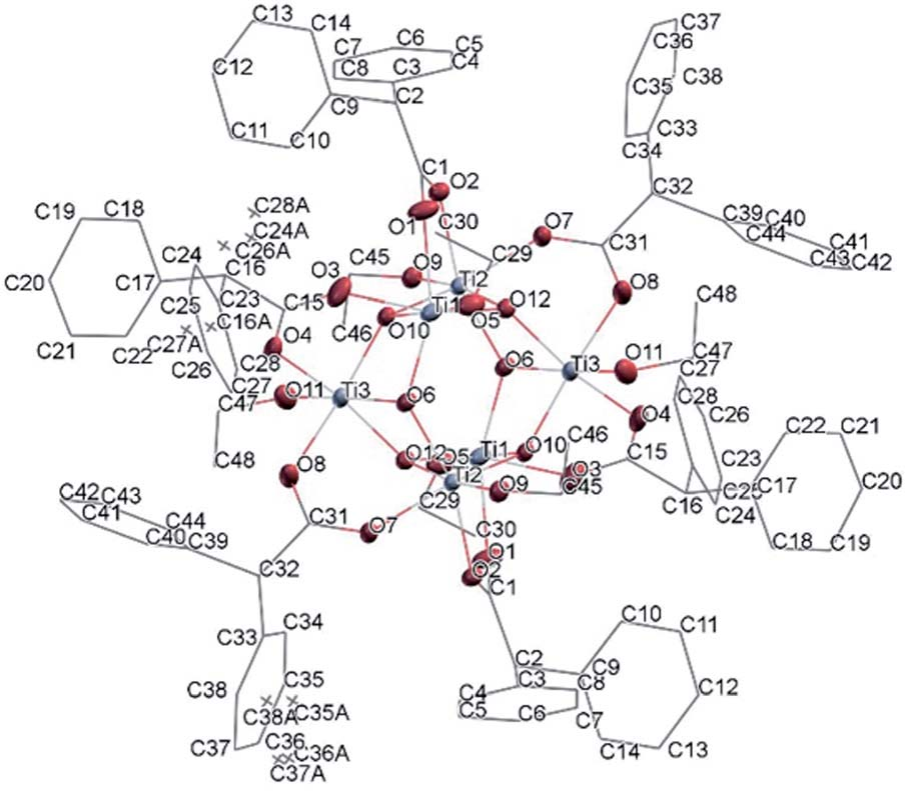
A view of the molecular structure of compound (III), with atom labelling. Displacement ellipsoids are drawn at the 50% probability level. H atoms have been omitted for clarity.

**Fig. 13:**
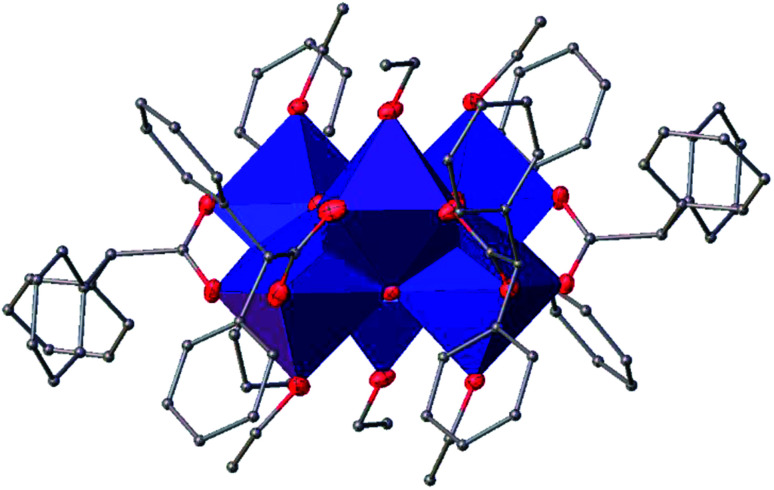
A polyhedral of (III), H atoms have been omitted for clarity.

**Fig. 14:**
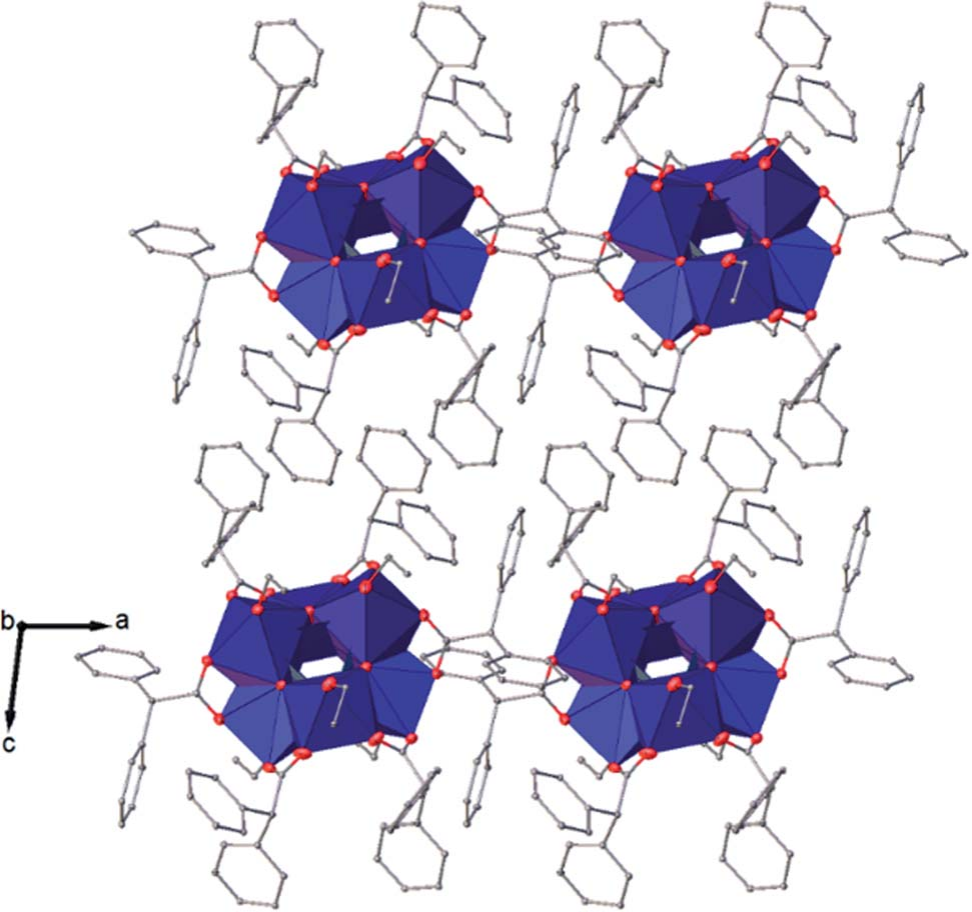
A view along the *b* axis of the crystal packing of compound (III).

The structure of Ti_6_O_6_(OEt)_6_(OOCCHPh_2_)_6_ was solved in *P*1̄ (Table SI1†). X-ray diffraction studies proved the formation of a hexameric structure surrounding the inversion centre with [Ti_3_O_3_(OEt)_3_(OOCCHPh_2_)_3_] moiety in the asymmetric unit (Fig. 12). The structure of (III) consists of two offset six-membered [Ti-(μ_3_-O)]_3_ rings joined through Ti–O bonds. The core arrangement is illustrated in Scheme 1c. This cluster has a condensation degree of 1 and a substitution degree of 1. From the literature and database searches it appears that this cluster type, with higher condensation degree, is one of the most common Ti_6_ type due to its robustness, while cores like the one of cluster (II), are uncommon.^23^ The Ti–O distances (Table SI4†) in μ_3_-oxide bridges change in the expected range 1.8845(15)–1.9163(15) Å and 2.1503(15)–2.16933(15) Å, and they are similar to the values observed in other hexanuclear Ti-oxo-carboxylato-alkoxo derivatives with general formula [Ti_6_(μ_3_-O)_6_(OR)_6_(OOCR′)_6_]. The hexanuclear μ_3_-oxo Ti(iv) core ({Ti_6_-(μ_3_-O)_6_}) adopts a hexagonal column geometry and is surrounded and stabilized by six carboxylate ligands on the equatorial plane and six ethoxide ligands along the vertical direction (Fig. 12). All Ti atoms in this complex adopt a six-fold octahedral environment (Fig. 13 and packing in Fig. 14). The Ti⋯Ti distances lie in the range 3.0915(7)–3.1333(6) Å (Table SI4†), comparable with values described in earlier reports.^5^

### Packing and Hirshfeld surface analyses

The packing of the three compounds was analysed, and Hirshfeld surfaces and fingerprint plots were generated on the refined structure models to help visualizing and studying geometry and intermolecular interactions within the crystals.

**Fig. 15:**
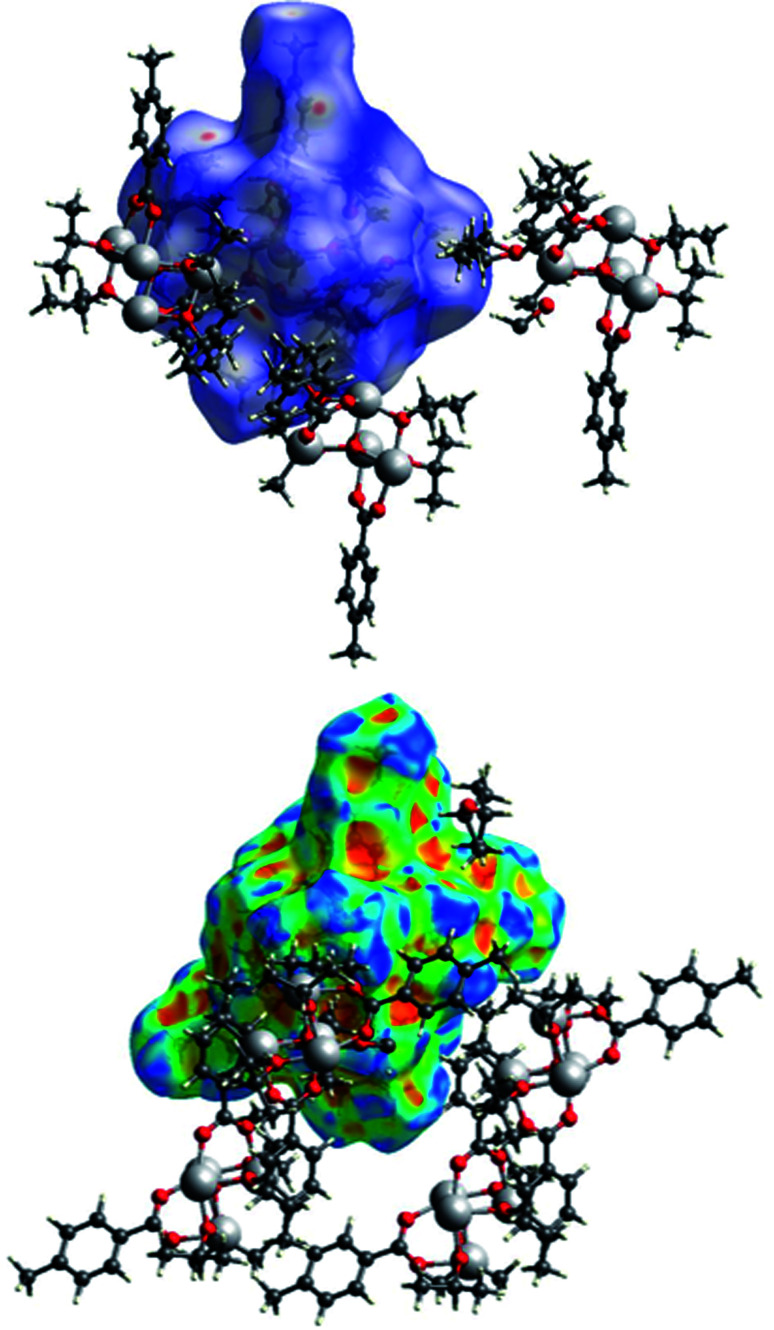
A view of the Hirshfeld surface of (I) with d-norm plotted (top) and shape index (bottom). Oxygen atoms are represented in red, carbon atoms in grey, hydrogen atoms in white, titanium atoms in light grey.

The Hirshfeld surface of (I) is shown in Fig. 15 with *d*_norm_ and shape index plotted upon it surrounded by some of the nearby molecules. In the top figure, the evident red spots indicate a short H⋯O interaction (on the right) and the equivalent O⋯H interaction (on the left of the picture). These are not proper hydrogen bonds since the hydrogen atom is bonded to a carbon atom and can be considered a weak hydrogen bond-like interaction. In the bottom figure the shape index is plotted on another view of the Hirshfeld surface. In the top right corner a C–H⋯π interaction with an OiPr group is evidenced by the presence of red and blue triangles.

**Fig. 16:**
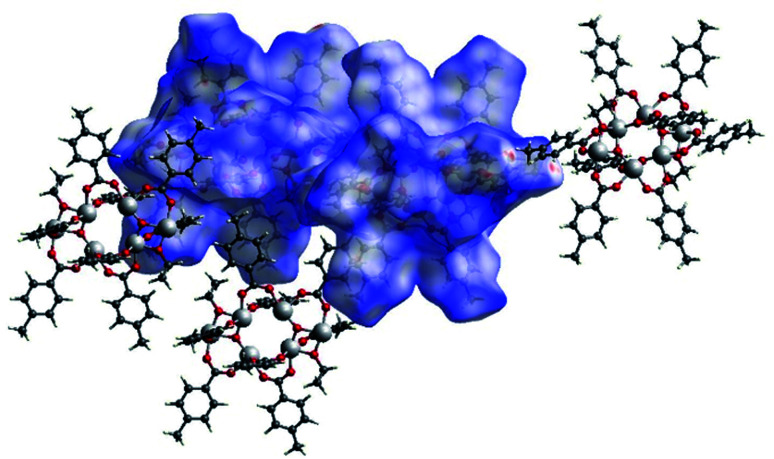
A view of the Hirshfeld surface of (II) with d-norm plotted.

In Fig. 16 the Hirshfeld surfaces of both independent molecules of (II) are represented. It is possible to see the flat areas in correspondence with the *p*-toluic ligand indicating the presence of π–π stacking. The different interactions between the two moieties are better evidenced by looking at the fingerprint plot and will be discussed in the related section.

**Fig. 17:**
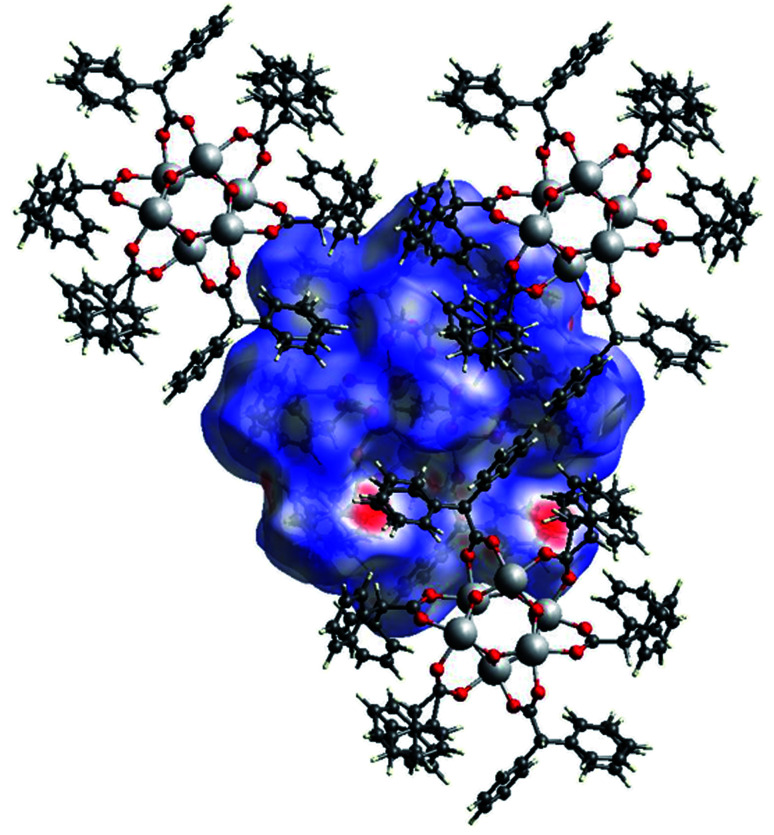
A view of the Hirshfeld surface of (III) with d-norm plotted. The large red spots indicate hydrophobic interactions C⋯C and C⋯H. The smaller red spot near to the large spots are O⋯HC interactions.

The Hirshfeld surface of (III) (Fig. 17) is characterized by large red areas on the *d*_norm_ plot indicating short contacts and are related to the aromatic groups' interactions. Nearby some smaller and lighter spots are visible in correspondence of O⋯HC interactions.

### Analysis of the fingerprint plots

In Fig. 19, the fingerprint plots of the three compounds are shown and the different interactions filtered. The fingerprint plots are symmetrical on the diagonal axis when there is only one molecule in the asymmetric unit because all interactions are reciprocal between two parts of the same molecule. In the case of (II) there are two independent molecules therefore there can be interactions between symmetry equivalent molecules but also between the two molecules and this makes the fingerprint plot asymmetric. Therefore, the asymmetry of the fingerprint plot can be interpreted as an indicator of the contacts between two non-equivalent molecules, confirming the reliability of the chosen space group, as before discussed.

**Fig. 18:**
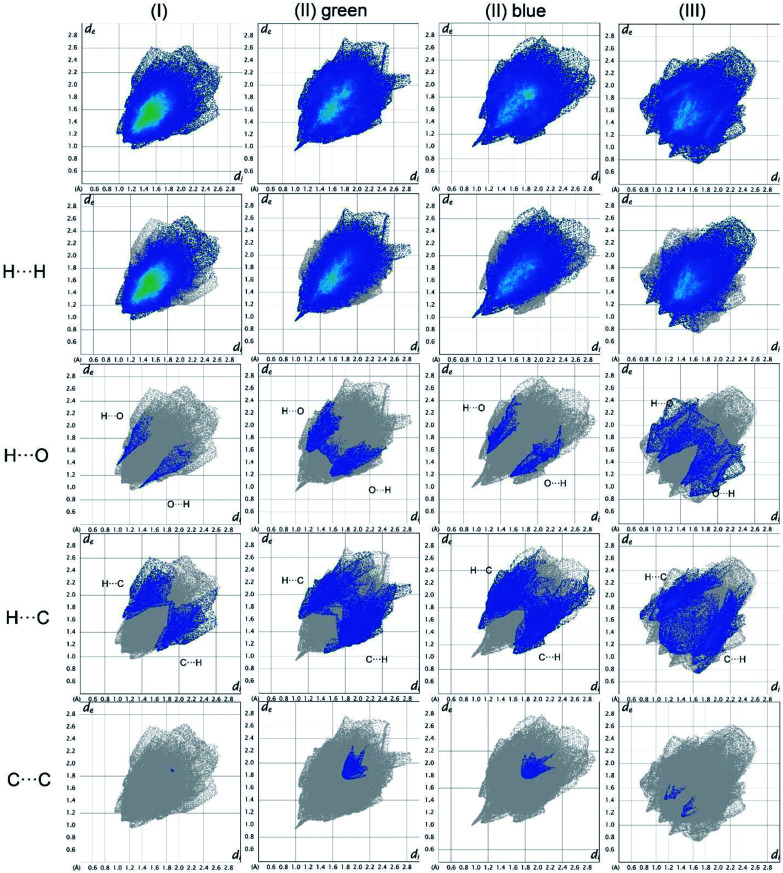
Hirshfeld fingerprint plots for (I), (II), (III) in columns. In rows total and filtered by element plots. Interactions between different elements are labelled in the plot.

**Fig. 19:**
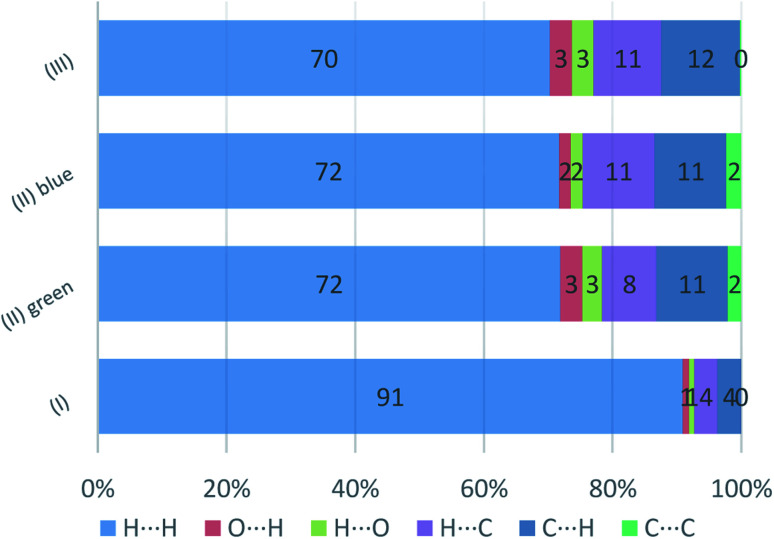
Percentage contributions to the Hirshfeld surface area for the intermolecular contacts in (I) (II), and (III). In the labels the first element is the one internal to the surface.

The presence of pairs of spikes pointing toward the bottom left corner of the fingerprint plot highlights the shortest contacts. In particular, hydrogen bonding, if present, is represented by two long spikes. Conversely, the presence of CH⋯π interactions is shown by lateral “wings” in the plot. In the same way, scattered points at high distances gives an idea of the closeness of the packing. The more the fingerprint plot is dispersed towards the top right corner, the more atoms are surrounded by “empty space”. The packing of all three compounds is mainly driven by hydrophobic interactions, because of the clustering of COO^−^ moieties towards Ti atoms. The graph in Fig. 18 allows to see the amount of surface involved in each kind of interaction. The percentages are not comparable one to each other in absolute terms because the total Hirshfeld surface is quite different between the three compounds, as can be seen from Table SI5 in the ESI file.† In our case there are some O⋯HC interactions (third row in Fig. 19) that are weaker than regular hydrogen bonds therefore the spikes are not pronounced and can be seen only in the filtered plots since they do not exceed the perimeter of the plot. These kinds of interactions are closer in (I) while in (III) there are two kinds of O⋯HC interactions since the filtered plot does not show a single spike but multiple triangular shapes.

## Experimental

### Materials and methods

All the reagents and solvents were commercially available and used as received. The cluster were synthesized following a procedure similar to the one described by Hong *et al.*^2^

### Synthesis of Ti_4_O_2_(O^i^Pr)_10_(OOCPhMe)_2_ (I)

*p*-Toluic acid (0.41 g, 3 mmol) was dissolved in 2-propanol (30 ml). The mixture was stirred at room temperature and titanium(iv) isopropoxide (2.7 ml, 9 mmol) were added. The resultant solution was introduced into a Parr Teflon-lined stainless-steel vessel (30 ml). The vessel was sealed and heated at 90 °C. The temperature was held for 2 days and then the mixture was left to cool to room temperature to obtain colourless crystals. The crystalline product was filtered off, washed with 2-propanol and dried at room temperature, yield 0.67 g (27%).

### Synthesis of Ti_6_O_4_(OEt)_8_(OOCPhMe)_8_ (II)

*p*-Toluic acid (5 g, 37 mmol) was suspended in 30 ml of EtOH in a beaker under stirring and mild warming. Then 1.2 ml (4 mmol) of titanium(iv) isopropoxide were added dropwise while stirring and the resulting clear solution was transferred to a Teflon reactor. The reactor was sealed and kept for 24 hours at 80 °C and then left to cool at room temperature for three days to allow crystal growth and colourless crystals were obtained. After filtering the crystals, the residual *p*-toluic acid was washed away with ethanol (5 × 20 ml) and the crystals were collected for measuring after drying at room temperature, yield 0.76 g (63%)

### Synthesis of Ti_6_O_6_(OEt)_6_(OOCCHPh_2_)_6_ (III)

Diphenylacetic acid (8.4 g, 40 mmol) was suspended in 30 ml of EtOH. The mixture was stirred at 50 °C and titanium(iv) isopropoxide (1.2 ml, 4 mmol) were added. The resultant clear solution was introduced into a Parr Teflon-lined stainless-steel vessel (30 ml). The vessel was sealed and heated for 2 days at 90 °C. The reactor was left to cool to room temperature to obtain near colorless crystals. The crystalline product was filtered off, washed with EtOH and dried at room temperature, yield 0.78 g (61%).

### Spectroscopic analysis details

The FTIR spectra were measured for crystalline compounds in the range of 4000–400 cm^−1^ with a Nicolet iS50 spectrometer equipped with the Specac Quest single-reflection diamond attenuated total reflectance (ATR) accessory. Spectral analysis was controlled by the OMNIC software package.

Diffuse Reflectance UV-Vis (DR UV-Vis) spectra were recorded using a Perkin Elmer Lambda 900 spectrometer equipped with a diffuse reflectance sphere attachment using BaSO_4_ as reference. The samples were grinded in a mortar and placed as loose powder in a 5 mm cuvette.

The ^1^H NMR spectra were obtained in DMSO-d6 with a Bruker Avance III HD 400 MHz spectrometer using standard pulse sequences.

### X-ray diffraction experiment details

Single crystal diffraction data were recorded with an Oxford Xcalibur CCD area detector (Oxford Diffraction, Abingdon-on-Thames, United Kingdom) diffractometer equipped with a Sapphire 3 CCD detector and a STOE IPDS 2T diffractometer, using graphite monochromatized Mo–K_(*λ* = 0.71069 Å). Numerical absorption correction was performed after the optimization of the crystal-shape description with the software HABITUS by Herrendorf and Bärnighausen.^35^ Structure solution and refinement were performed using SHELXT 2014/5 (ref. 36) and *SHELXL 2016/6*.^37^*ORTEP*^38^ for Windows and CCDC Mercury^39^ were used for visualizing structures, *WinGX*^38^ publication routines were used for the crystallographic data files.

Crystal data, data collection and structure refinement details for (I)–(III) are summarized in Table SI1.† All non-hydrogen atoms were refined anisotropically. Hydrogen atoms were refined in geometrically idealized position with isotropic temperature factors 1.2 times the equivalent isotropic temperature factors *U*_eq_ of their attached atoms (1.5 for CH_3_ groups). Three ^i^Pr groups in (I) were found disordered in two positions: C32–C34 (s.o.f. of 0.556(10) and 0.444(10)), C35–C37 (s.o.f. of 0.409(15) and 0.591(15)) and C44–C46 (s.o.f. of 0.358(11) and 0.642(11)). Two Et groups in (II) show disorder over two positions: C39–C40 (s.o.f. of 0.383(12) and 0.617(12)) and C73–C74 (s.o.f. of 0.36(2) and 0.64(3)). The three –Ph–CH_3_ groups in (II) must have been modelled as disordered: C1–C7 (s.o.f. of 0.282(9) and 0.718(9)) C41–C47 (s.o.f. of 0.755(5) and 0.245(5)) and C57–C63 (s.o.f. of 0.512(10) and 0.488(10)). Disordered C atoms in Ph rings in (III): C16 (s.o.f. of 0.798(8) and 0.202(8)) C24, C26–C28 (s.o.f. of 0.797(4) and 0.203(4)) and C36–C38 (s.o.f. of 0.748(11) and 0.252(11)). Displacement ellipsoids of the carbon atoms tend to achieve very high values so EADP SHELX instruction was applied to constrain them.

XRPD analysis was performed on a Bruker D8 Advance diffractometer with a Lynx-Eye XE-T detector and Cu kα radiation source (*λ* = 0.15418 nm). The instrument is equipped with an auto-sampler with nine positions, rotating sample holders and an air scatter knife. On both the primary and secondary optics Soller slits 2.5° opening were positioned. Goniometer radius is 280 mm. The tube was set at 40 mA in current and 40 kV in electric potential. The patterns were collected in Bragg–Brentano geometry from 3° to 70° 2*θ* with a step-size of 0.01° and exposure time of 0.05 s, automatic divergence slits were set to obtain constant sample illumination of 17 mm.

All samples were grinded in a mortar and measured without sample rotation. Compound (III) was also measured without grinding but rotating the sample-holder to reduce preferred orientation effects. TOPAS 5 Academic^40^ was used to perform the Rietveld refinements of the XRPD patterns, using the single crystal structures.

### Thermal analysis

A TA Instruments SDT Q600 was used to collect simultaneous TGA/DSC data under inert atmosphere in N_2_ with a temperature ramp of 20 °C min^−1^ from 50 to 600 °C.

### Microscopy

A Zeiss STEMI 508 microscope with 2× frontal optics, Schott VisiLED ring light and a 20 MPx SONY sensor camera was used to collect optical images with high resolution.

SEM images at different magnification were recorded using a Hitachi FLEXSEM 1000 with tungsten filament as the electron source at 15 kV. The samples were coated with a graphite layer to prevent surface charging.

### Hirshfeld analyses

Hirshfeld surfaces were generated by the program Crystal Explorer 17.5.^41^ The parameter *d*_i_ represents the distance from the surface to the nearest atom interior to the surface while *d*_e_ is the distance from the surface to the nearest atom exterior to the surface. Taking these (*d*_i_, *d*_e_) pairs and normalizing them with respect to the van der Waals radii of their corresponding atoms results in *d*_norm_. When *d*_norm_ is plotted on the Hirshfeld surface, contacts shorter than the sum of the van der Waals radii of the two atoms are being highlighted in red, contacts close in length to the van der Waals limit are in white, while the blue colour represents longer contacts. The fingerprint plot is obtained by plotting (*d*_e_, *d*_i_) pairs for each point of the Hirshfeld surface. The colour of the points (ranging from blue, green, yellow, to red) represents the frequency of occurrence of the interaction. The analysis of the fingerprint plot allows to easily investigate the intermolecular interactions filtering the contribution from each feature and visualizing the weight of the interaction in driving the packing.

## Summary and conclusions

One tetranuclear and two hexanuclear novel Ti-oxo clusters were synthesized and characterized by different techniques. The synthesis was made by an easy solvothermal method that can be tuned to obtain different cores by changing the reaction stoichiometry and dilution, and to obtain different clusters by changing the ligands. The high purity of the products was demonstrated by FT-IR spectroscopy and XRPD analysis. Only compound III resulted less homogeneous and not stable under grinding. Moreover, it shows a more complex UV spectrum, with an absorption in the visible range. Optical and electron microscopy indicated that III is a mixture of large crystallites with powders, indeed of the same nature as indicated by XRPD. TGA/DSC indicated similar decomposition behaviours, but with differences in the onset temperatures and in the tendency to form carbonaceous species, due to the different cluster structures and stabilities. The structures of I–III were solved by single crystal X-ray diffraction and the observed cluster structures and packings carefully examined. The packing interactions in the three clusters were analysed using the Hirshfeld surfaces method. The packings were mainly driven by C–O⋯H bonds, that can be described as weak hydrogen bonds, and by hydrophobic interactions. The structure of the Ti_6_O_4_(OEt)_8_(OOCPhMe)_8_ cluster has the peculiarity of being formed by two half molecules in the asymmetric unit forming two moieties that have very similar bond lengths and angles. The difference between these two molecules is thus only given by their mutual interactions.

## Conflicts of interest

There are no conflicts to declare.

## Supplementary Material

RA-011-D0RA09691A-s001

RA-011-D0RA09691A-s002
