# Supplementary material for: Solvothermal synthesis and structural characterization of three polyoxotitanium-organic acid clusters
Source: RSC Adv. 2021 Jul 23;11(40):25068–78. doi: 10.1039/d0ra09691a (PMC9037011; doi:10.1039/d0ra09691a)
Supplement: RA-011-D0RA09691A-s001 [file RA-011-D0RA09691A-s001.pdf]

Electronic Supplementary Material (ESI) for RSC Advances.
This journal is © The Royal Society of Chemistry 2021

Supplementary information

Solvothermal synthesis and structural characterization of three polyoxotitanium-organic acid clusters

Katarzyna Kazimierczuk,[a] Marco Milanesio,[b] Anna Dołęga,[a] Luca Palin,[b,c] Maja Walencik,[a] Michał Jurkowski[a] and Eleonora Conterosito,[b*]

[a.] *Department of Inorganic Chemistry, Chemical Faculty, Gdańsk University of Technology, Narutowicza St. 11/12, 80-233 Gdańsk, Poland*
[b.] *Dipartimento di Scienze e Innovazione Tecnologica, Via T. Michel 11, 15121 ALESSANDRIA, Italy *email: eleonora.conterosito@uniupo.it.*
[c.] *Nova Res s.r.l., Via D. Bello 3, 28100 Novara, Italy; https://www.novares.org*

	(I)	(II)	(III)
Crystal data			
Chemical formula	$C_{46}H_{84}O_{16}Ti_4$	$C_{80}H_{96}O_{28}Ti_6$	$C_{96}H_{96}O_{24}Ti_6$
M_r	1084.73	1792.96	1921.12
Crystal system, space group	Monoclinic, $P2_1/n$	Triclinic, *P-1*	Triclinic, *P-1*
Temperature (K)	120	120	120
a, *b*, *c* (Å)	12.216 (6), 19.637 (5), 23.02 (1)	14.942 (2), 15.493 (2), 20.120 (3)	12.190 (2), 13.515 (2), 15.260 (3)
α, β, γ (°)	90, 99.56 (4), 90	72.82 (1), 83.87 (1), 78.35 (1)	107.57 (13), 92.10 (1), 105.43 (1)
V ($Å^3$)	5446 (4)	4352 (1)	2291.4 (7)
Z	4	2	1
Radiation type	Mo *K*α	Mo *K*α	Mo *K*α
μ (mm^{-1})	0.63	0.6	0.57
Crystal size (mm^3)	0.32 × 0.25 × 0.17	0.15 × 0.13 × 0.1	0.35 × 0.17 × 0.14
Data collection			
Absorption correction	Empirical		
T_{min}, T_{max}	0.845, 0.930	0.929, 0.958	0.864, 0.932
No. of measured, independent and observed [$I > 2\sigma(I)$] reflections	28207, 10598, 8156	33376, 16867, 9996	16107, 8379, 7358
R_{int}	0.033	0.047	0.023
$(\sin \theta/\lambda)_{max}$ ($Å^{-1}$)	0.617	0.617	0.606
Refinement			
$R[F^2 > 2\sigma(F^2)]$, $wR(F^2)$, S	0.051, 0.141, 1.03	0.055, 0.147, 1.02	0.038, 0.103, 1.05
No. of reflections	10598	16867	8379
No. of parameters	649	1254	652
No. of restraints	4	14	0
H-atom treatment	H atoms treated by a mixture of independent and constrained refinement	H-atom parameters constrained	H atoms treated by a mixture of independent and constrained refinement
$\Delta\rho_{max}$, $\Delta\rho_{min}$ (e $Å^{-3}$)	1.34, −0.51	0.53, −0.63	0.84, −0.58

Table SI1. Experimental details for the three compounds.

Distances (Å)					
Ti-(μ_2-O)			Ti-(O^{i}Pr)		
	Ti(1)- O(6)	1.840(2)		Ti(1)-O(3)	1.790(2)
	Ti(4)- O(6)	1.846(2)		Ti(2)-O(8)	1.805(2)
Ti-(μ_4-O)				Ti(2)-O(16)	1.783(2)
	Ti(1)-O(7)	2.058(2)		Ti(3)-O(11)	1.789(2)
	Ti(2)-O(7)	2.075(2)		Ti(3)-O(12)	1.803(2)
	Ti(3)-O(7)	2.042(2)		Ti(4)-O(15)	1.789(2)
	Ti(4)-O(7)	2.0560(19)	Ti-(OOCPhMe)		
				Ti(1)-O(1)	2.146(2)
				Ti(2)-O(9)	2.046(2)
Ti-(μ-O^{i}Pr)				Ti(3)-O(2)	2.061(2)
	Ti(1)-O(4)	2.023(2)		Ti(4)-O(14)	2.171(2)
	Ti(3)-O(13)	2.034(2)	Ti---Ti		
	Ti(4)-O(10)	1.960(2)		Ti(1)-Ti(4)	2.9565(17)
	Ti(4)-O(13)	2.008(2)		Ti(1)-Ti(3)	3.1562(12)
				Ti(1)-Ti(2)	3.2187(12)
				Ti(2)-Ti(4)	3.1699(11)
				Ti(3)-Ti(4)	3.1977(13)
Angles (º)					
Ti-(μ_2-O)-Ti			Ti-(μ- O^{i}Pr)-Ti		
	Ti(1)-O(6)-Ti(4)	106.66(10)		Ti(2)-O(4)-Ti(1)	105.49(9)
				Ti(1)-O(5)-Ti(3)	101.63(9)
Ti-(μ_4-O)-Ti				Ti(4)-O(10)-Ti(2)	102.94(9)
	Ti(3)-O(7)-Ti(4)	102.56(9)		Ti(4)-O(13)-Ti(3)	104.61(9)
	Ti(3)-O(7)-Ti(1)	100.66(8)			
	Ti(4)-O(7)-Ti(1)	91.89(8)			
	Ti(3)-O(7)-Ti(2)	146.85(10)			
	Ti(4)-O(7)-Ti(2)	100.24(8)			
	Ti(1)-O(7)-Ti(2)	102.31(8)			

Table SI2. Bond lengths (Å) and angles (º) for **(I)**

Distances (Å)					
Ti-(μ_2-O)			Ti-(OOCPhMe)	Ti(3)-O(1)$^{\#1}$	2.122(4)
	Ti(1)-O(14)	1.754(2)		Ti(1)-O(2)	2.013(3)
	Ti(2)-O(14)	1.878(2)		Ti(1)-O(3)	2.047(3)
	Ti(4)-O(28)	1.760(3)		Ti(2)-O(4)	2.081(2)
	Ti(5)- O(28)	1.874(3)		Ti(1)-O(5)	2.003(3)
Ti-(μ_3-O)				Ti(2)-O(6)	2.084(3)
	Ti(1)-O(13)$^{\#1}$	1.894(2)		Ti(2)-O(7)	2.040(3)
	Ti(2)-O(13)	1.898(2)		Ti(3)-O(8)	2.042(3)
	Ti(3)-O(13)	2.103(2)		Ti(6)-O(15)$^{\#2}$	2.171(3)
	Ti(4)-O(26)	1.892(2)		Ti(4)-O(16)	1.996(3)
	Ti(5)-O(26)$^{\#2}$	1.910(2)		Ti(4)-O(17)	2.043(3)
	Ti(6)-O(26)$^{\#2}$	2.076(3)		Ti(5)-O(18)	2.084(3)
Ti-(μ-OEt)				Ti(5)-O(19)	2.095(3)
	Ti(3)-O(11)	1.961(3)		Ti(4)-O(20)	1.996(3)
	Ti(1)-O(11)$^{\#1}$	2.073(2)		Ti(5)-O(21)	2.044(3)
	Ti(6)-O(24)	1.958(3)		Ti(6)-O(22)	2.016(3)
	Ti(4)-O(24)$^{\#2}$	2.084(3)			
Ti-(OEt)			Ti---Ti		
	Ti(3)-O(9)	1.781(3)		Ti(1)-Ti(3)$^{\#1}$	3.1116(10)
	Ti(3)-O(10)	1.768(4)		Ti(1)-Ti(2)	3.3780(10)
	Ti(2)-O(12)	1.769(3)		Ti(4)-Ti(6)$^{\#2}$	3.1043(12)
	Ti(6)-O(23)	1.789(3)		Ti(4)-Ti(5)	3.3752(11)
	Ti(6)-O(25)	1.787(3)			
	Ti(5)-O(27)	1.754(3)			
Angles (º)					
Ti-(μ_2-O)-Ti			Ti-(μ-OEt)-Ti	Ti(3)-O(11)-Ti(1)$^{\#1}$	100.93(10)
	Ti(1)-O(14)-Ti(2)	136.88(15)		Ti(6)-O(24)-Ti(4)$^{\#2}$	100.31(11)
	Ti(4)-O(28)-Ti(5)	136.50(14)			
Ti-(μ_3-O)-Ti					
	Ti(1)$^{\#1}$-O(13)-Ti(2)	128.09(12)			
	Ti(1)$^{\#1}$-O(13)-Ti(3)	102.14(10)			
	Ti(2)-O(13)-Ti(3)	129.40(13)			
	Ti(4)-O(26)-Ti(5)$^{\#2}$	128.26(14)			
	Ti(4)-O(26)-Ti(6)$^{\#2}$	102.88(11)			
	Ti(5)$^{\#2}$-O(26)-Ti(6)$^{\#2}$	127.90(13)			

Table SI3. Bond lengths (Å) and angles (º) for **(II)**

Distances (Å)					
Ti-(μ_3-O)			Ti-($OOCHPh_2$)		
	Ti(1)-O(6)	1.8868(15)		Ti(1)-O(1)	2.0663(17)
	Ti(2)-O(6)	1.9068(15)		Ti(2)-O(2)$^{\#1}$	2.0672(16)
	Ti(3)-O(6)$^{\#1}$	2.1530(15)		Ti(1)-O(3)	2.0490(17)
	Ti(3)-O(10)	1.8845(15)		Ti(3)-O(4)$^{\#1}$	2.0648(17)
	Ti(2)-O(10)	1.9163(15)		Ti(2)-O(7)	2.0550(15)
	Ti(1)-O(10)$^{\#1}$	2.1633(15)		Ti(3)-O(8)$^{\#1}$	2.0795(16)
	Ti(3)-O(12)	1.8963(15)	Ti---Ti		
	Ti(1)-O(12)	1.9030(15)		Ti(1)-Ti(3)$^{\#1}$	3.0915(7)
	Ti(2)-O(12)$^{\#1}$	2.1503(15)		Ti(1)-Ti(2)$^{\#1}$	3.1333(6)
Ti-(OEt)				Ti(2)-Ti(3)$^{\#1}$	3.1130(8)
	Ti(1)-O(5)	1.7672(16)			
	Ti(2)-O(9)	1.7596(15)			
	Ti(3)-O(11)	1.7719(16)			
**Angles (º)**					
Ti-(μ_3-O)-Ti					
	Ti(1)-O(6)-Ti(2)	134.32(8)			
	Ti(1)-O(6)-Ti(3)$^{\#1}$	99.65(6)			
	Ti(2)-O(6)-Ti(3)$^{\#1}$	99.95(7)			
	Ti(3)-O(10)-Ti(2)	133.80(8)			
	Ti(3)-O(10)-Ti(1)$^{\#1}$	99.36(6)			
	Ti(2)-O(10)-Ti(1)$^{\#1}$	100.18(6)			
	Ti(3)-O(12)-Ti(1)	134.13(8)			
	Ti(3)-O(12)-Ti(2)$^{\#1}$	100.39(7)			
	Ti(1)-O(12)-Ti(2)$^{\#1}$	101.08(6)			

Table SI4. Bond lengths (Å) and angles (º) for **(III)**

	(I)	(II) green	(II) blue	(III)
	(%)	(%)	(%)	(%)
H···H	90.9	71.8	71.8	70.1
O···H	1.0	3.4	1.8	3.4
H···O	0.8	3.1	1.8	3.4
H···C	3.6	8.4	11.2	10.5
C···H	3.7	11.1	11.2	12.2
C···C	0.0	2.1	2.3	0.2
Area	795.77	1306.87	1299.52	1247.76
Volume	1350.6	2139.72	2180.18	2277.33
Globularity	0.743	0.614	0.626	0.671
Asphericity	0.031	0.039	0.034	0.043

Table SI5. Hirshfeld analysis data for (I), (II) both moieties and (III)

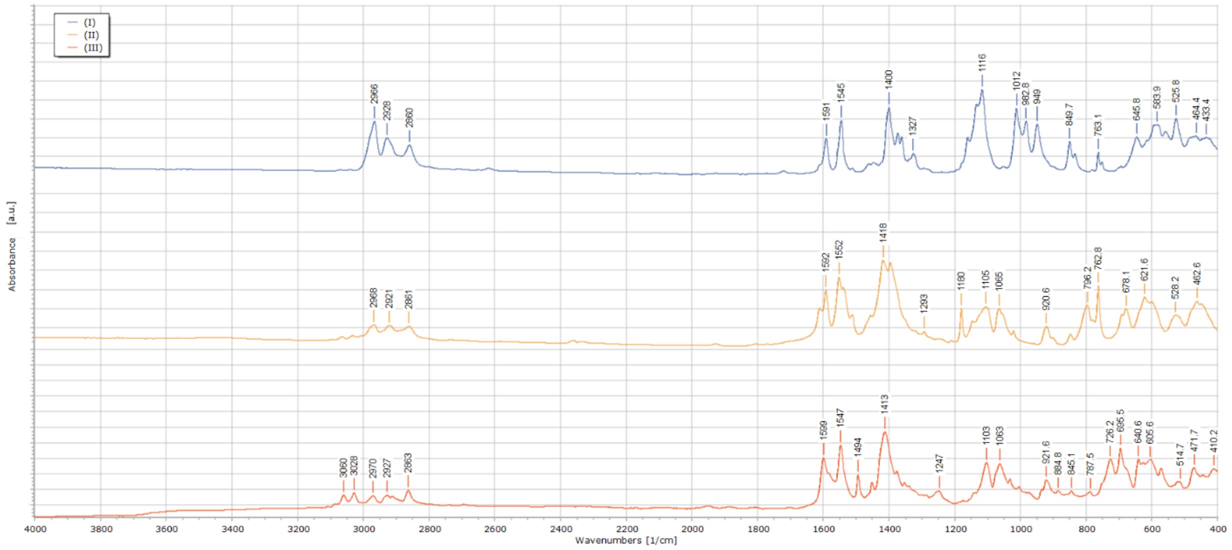

Figure SI1. FTIR spectra of **(I)** (top), **(II)** (middle) and **(III)** (bottom) with labels.

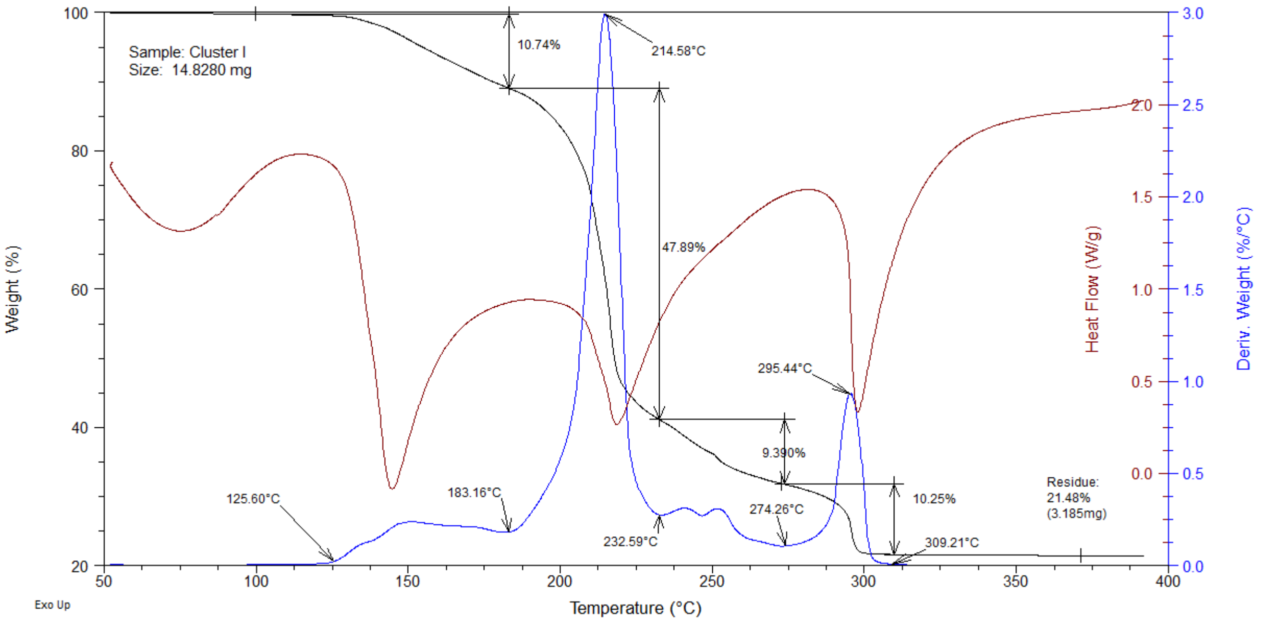

Figure SI2. TGA-DSC plot of (I)

**Cluster (I)**	$C_{46}H_{84}O_{16}Ti_4$	$Ti_4O_2(OiPr)_{10}(OOCPhMe)_2$		
	mass per moiety (u)	number of moieties	mass (u)	% mass
iPrO	59.088	5	295.44	27.239
iPrO	59.088	5	295.44	27.239
OOCPhMe	135.142	2	270.284	24.919
Ti4O2			223.466	**20.603**
		total mass (u)	1084.63	
		experimental % mass residue		**21%**

Table SI6. Mass calculations for TGA of (I)

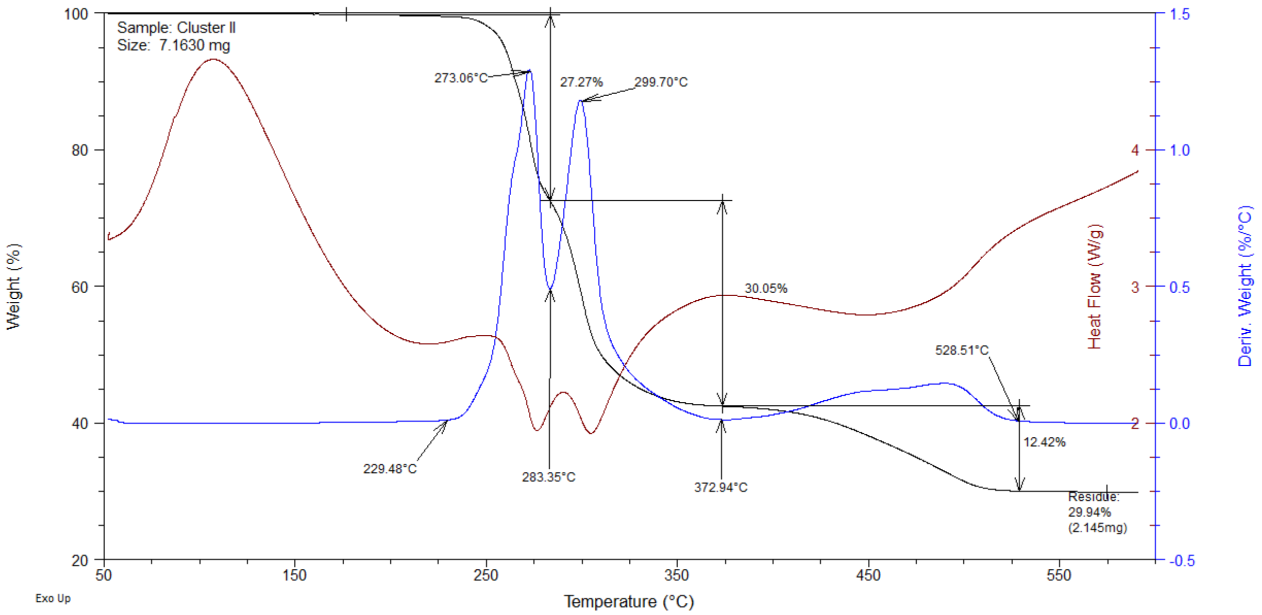

Figure SI3. TGA-DSC plot of (II)

**Cluster (II)**	$C_{80}H_{96}O_{28}Ti_6$	$Ti_6O_4(OEt)_8(OOCPhMe)_8$		
	mass per moiety (u)	number of moieties	mass (u)	% mass
OEt	45.061	8	360.488	20.107
OOCPhMe	135.142	8	1081.136	60.304
Ti6O4			351.198	19.589
TiO2	79.865	6	479.19	**26.728**
		total mass (u)	1792.822	
		experimental % mass residue		**30%**

Table SI7. Mass calculations for TGA of (II)

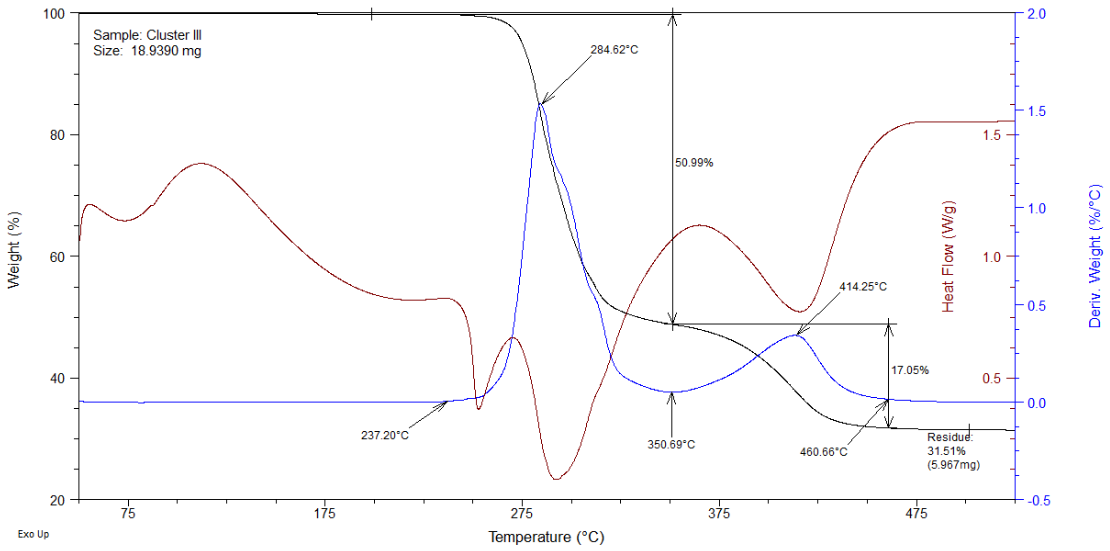

Figure SI4. TGA-DSC plot of (III)

**Cluster (III)**	$C_{96}H_{96}O_{24}Ti_6$	$Ti_6O_6(OEt)_6(OOCCHPh_2)_6$		
	mass per moiety (u)	number of moieties	mass (u)	% mass
OEt	45.061	6	270.366	14.074
OOCCHPh2	211.24	6	1267.44	65.978
Ti6O6			383.196	19.948
TiO2	79.865	6	479.19	**24.945**
		total mass (u)	1921.002	
		experimental % mass residue		**31%**

Table SI8. Mass calculations for TGA of (III)

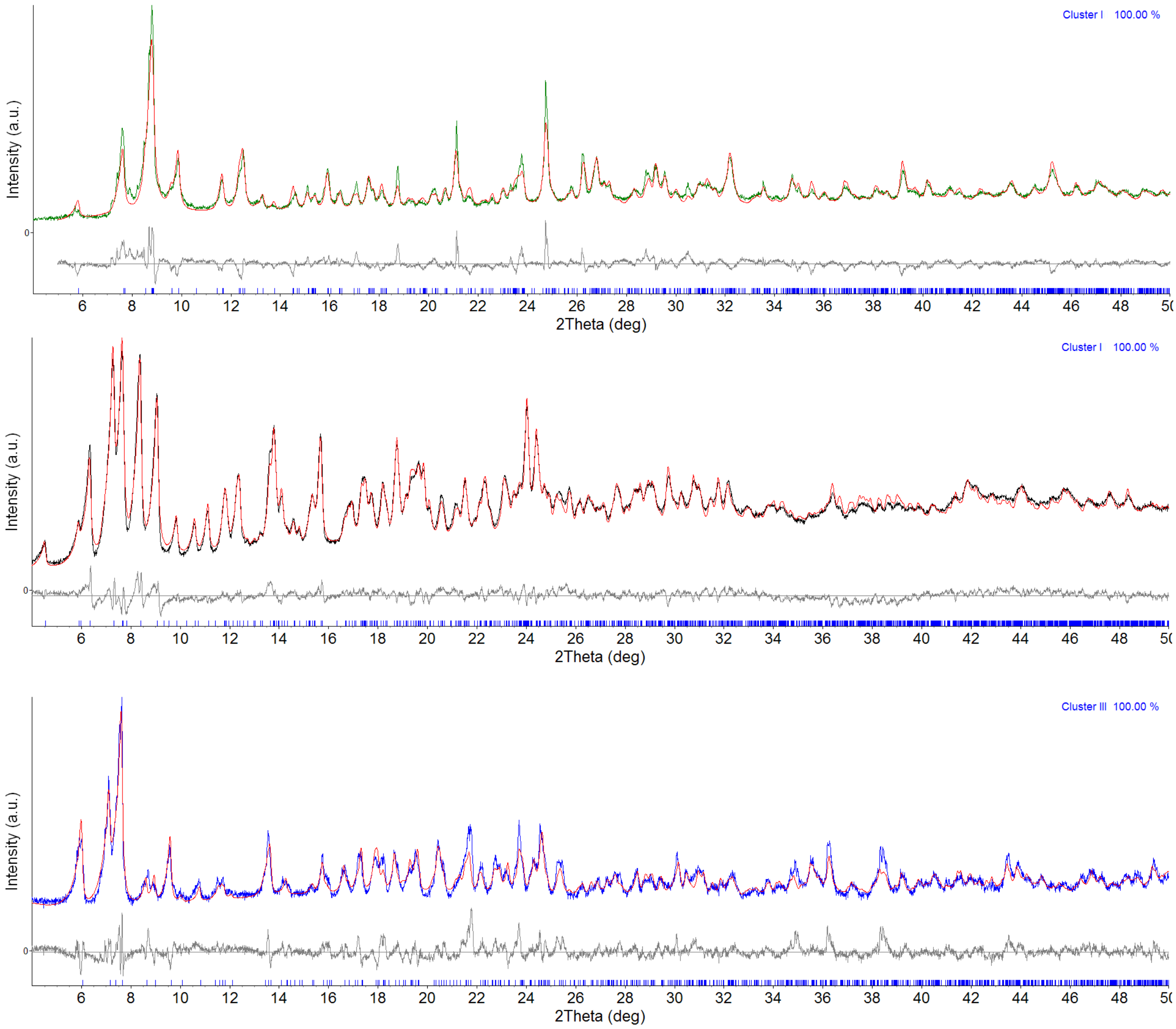

Figure SI5. XRPD pattern of cluster I (top - green), cluster II (middle - black) and cluster III (bottom - blue). Rietveld fit in red and difference curve in grey.

Cluster I	Cluster II	Cluster III
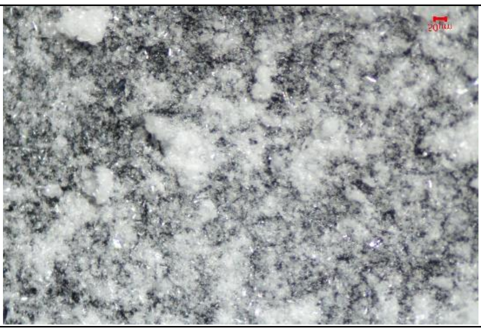	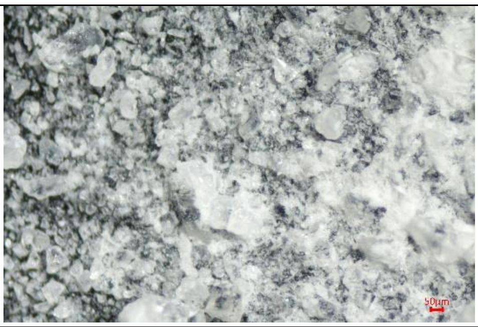	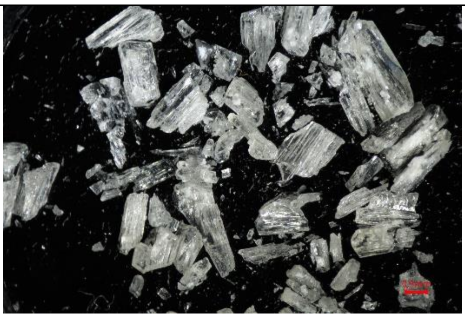
Optical microscopy 50x		
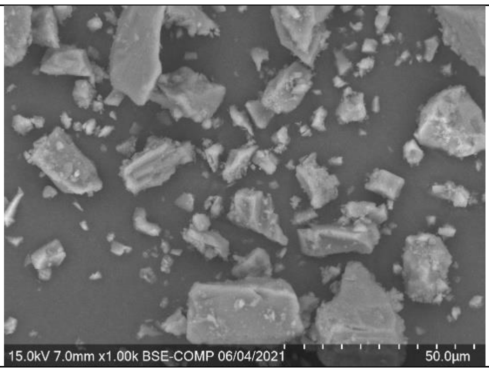	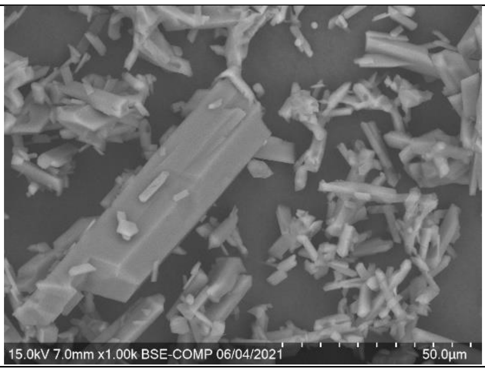	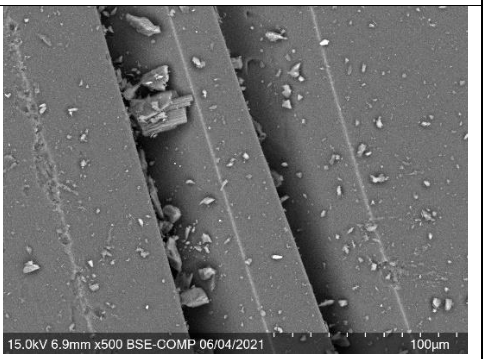
SEM BSE – 1000 x (500 for cluster III with larger crystallites)		
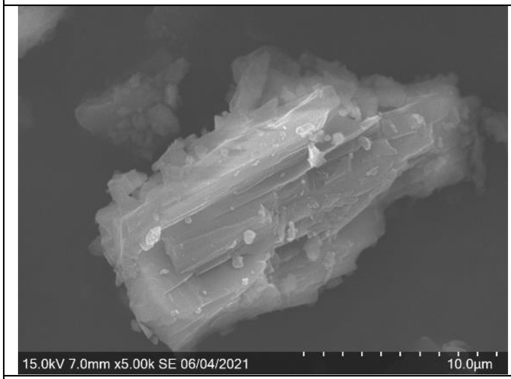	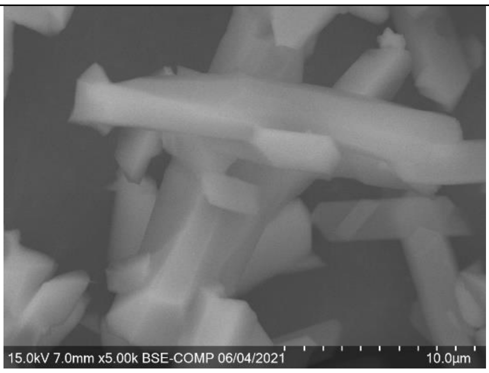	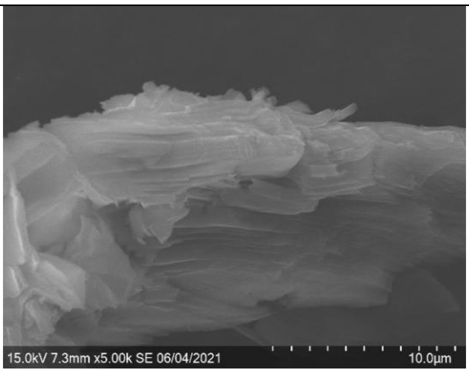
SEM SE – 5000x		

Figure SI6. Optical and electron microscopy images of cluster I, II and III at low, medium and high magnification

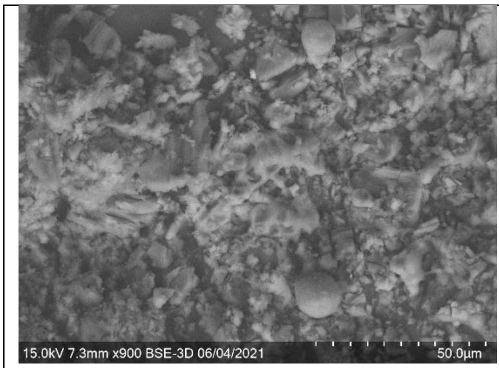	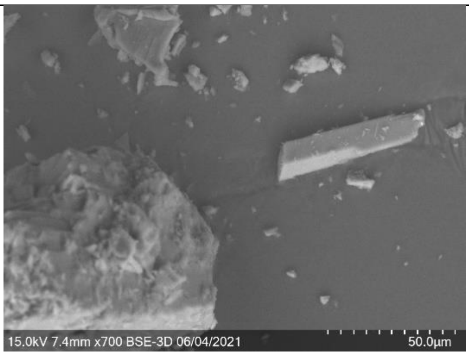	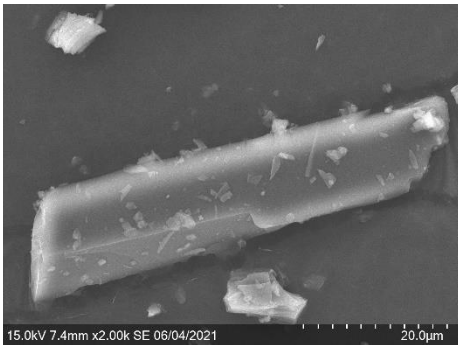
500 x before grinding	700x after grinding	2000x after grinding

Figure SI7. SEM images of cluster III after grinding, highlighting the co-existence of different morphologies.

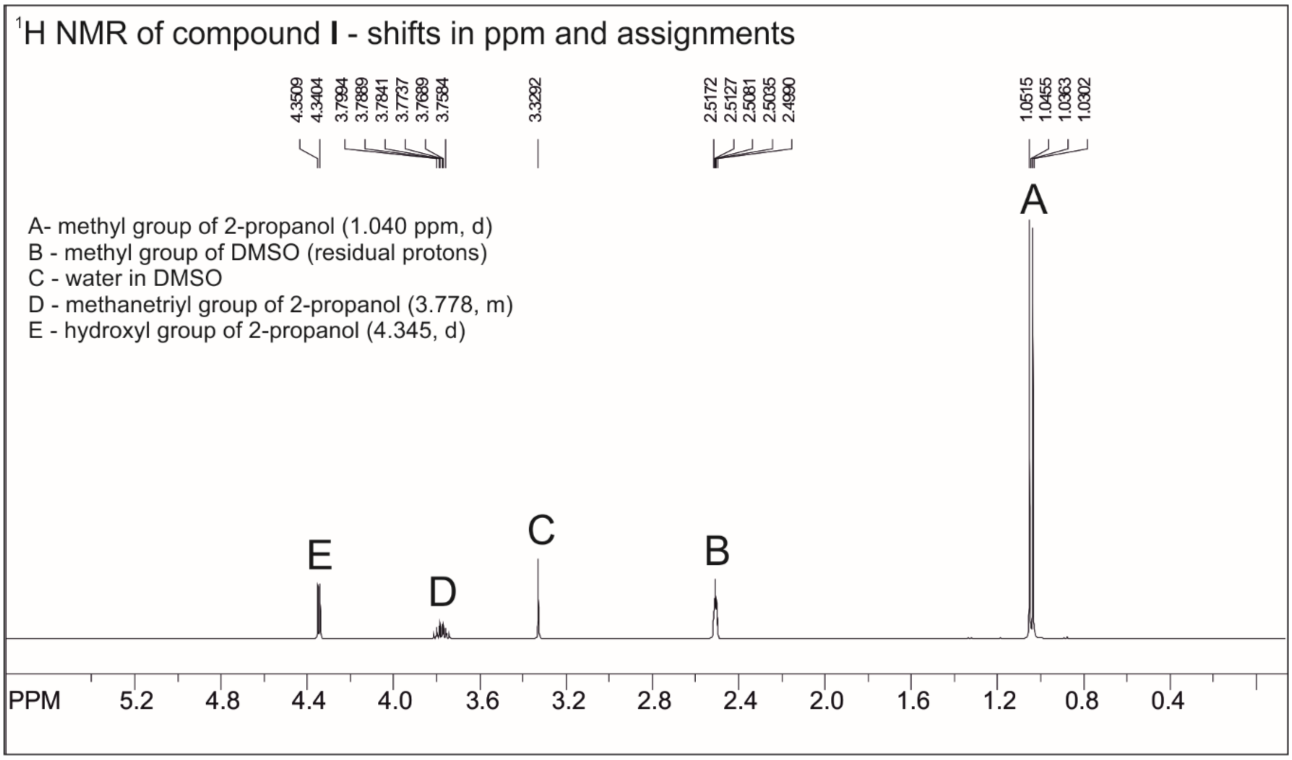

Figure SI8. ^{1}H NMR spectra of complex **I** – shifts and assignments.

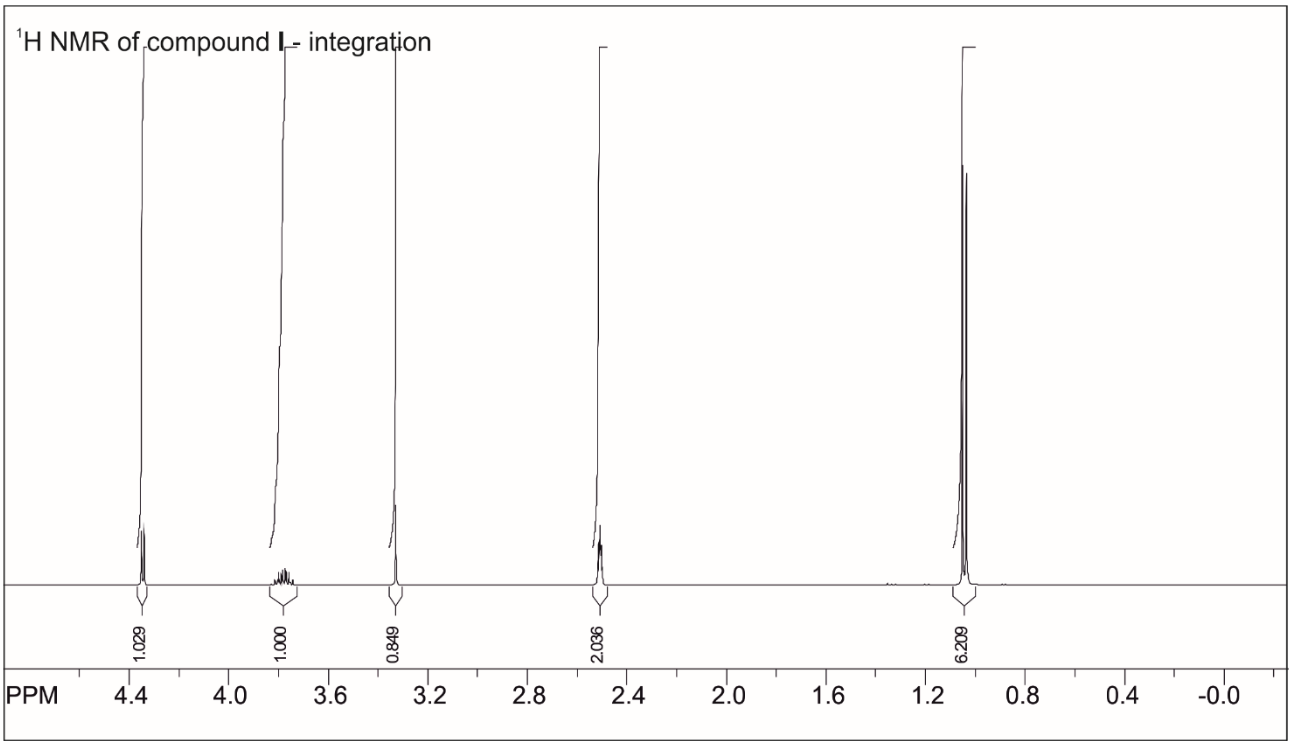

Figure SI9. ^{1}H NMR spectra of complex **I** – integration
